# Early Parenteral Nutrition in Patients with Biliopancreatic Mass Lesions, a Prospective, Randomized Intervention Trial

**DOI:** 10.1371/journal.pone.0166513

**Published:** 2016-11-18

**Authors:** Janine Krüger, Peter J. Meffert, Lena J. Vogt, Simone Gärtner, Antje Steveling, Matthias Kraft, Julia Mayerle, Markus M. Lerch, Ali A. Aghdassi

**Affiliations:** 1 Department of Medicine A, University Medicine Greifswald, Greifswald, Germany; 2 Institute for Community Medicine, University Medicine Greifswald, Greifswald, Germany; 3 cts Vinzentius Krankenhaus, Landau, Germany; Emory University Winship Cancer Institute, UNITED STATES

## Abstract

**Purpose:**

Patients with biliopancreatic tumors frequently suffer from weight loss and cachexia. The in-hospital work-up to differentiate between benign and malignant biliopancreatic lesions requires repeated pre-interventional fasting periods that can aggravate this problem. We conducted a randomized intervention study to test whether routine in-hospital peripheral intravenous nutrition on fasting days (1000 ml/24 h, 700 kcal) has a beneficial effect on body weight and body composition.

**Material and Methods:**

168 patients were screened and 100 enrolled in the trial, all undergoing in-hospital work-up for biliopancreatic mass lesions and randomized to either intravenous nutrition or control. Primary endpoint was weight loss at time of hospital discharge; secondary endpoints were parameters determined by bioelectric impedance analysis and quality of life recorded by the EORTC questionnaire.

**Results:**

Within three months prior to hospital admission patients had a median self-reported loss of 4.0 kg (25*th: -10.0 kg and 75*th* percentile: 0.0kg) of body weight. On a multivariate analysis nutritional intervention increased body weight by 1.7 kg (95% CI: 0.204; 3.210, p = 0.027), particularly in patients with malignant lesions (2.7 kg (95% CI: 0.71; 4.76, p < 0.01).

**Conclusions:**

In a hospital setting, patients with suspected biliopancreatic mass lesions stabilized their body weight when receiving parenteral nutrition in fasting periods even when no total parenteral nutrition was required. Analysis showed that this effect was greatest in patients with malignant tumors. Further studies will be necessary to see whether patient outcome is affected as well.

**Trial Registration:**

ClinicalTrials.gov NCT02670265

## Introduction

In industrialized countries, pancreatic cancer is the third most common cause of cancer death and its proportion is rising [1]. Pancreatic cancer is an aggressive tumor characterized by early metastasis formation and late diagnosis. The five-year survival rate is 8% and the median survival time six months [2,3]. Almost all patients with pancreatic cancer are at a high risk losing weight, suffering from malnutrition and developing the most severe degrees of cachexia [4–7]. More than one third of patients lose >10% of their pre-illness stable body weight and have experienced a significant weight loss at time of diagnosis [8–10]. Up to 80% of patients develop cachexia during their illness [11–16]. Cancer cachexia is a multifactorial syndrome characterized by severe body weight, fat and muscle loss and an increased protein catabolism as a result of an underlying malignant disease [17]. The patients often suffer from absence of appetite, abdominal pain, diarrhea, anorexia, back pain, fatigue, jaundice, diabetes, maldigestion and systemic inflammation [13,18]. The same holds true for biliary tract cancer [19]. Furthermore, there are changes in metabolism by an increased protein catabolism and energy expenditure [20]. The combination of all of these symptoms is usually called “cancer anorexia-cachexia syndrome” [13]. The patients with malnutrition or cachexia have a poorer prognosis than patients with stable weight and body composition [12,21]. Several studies showed that malnutrition leads to skeletal muscle wasting and fat degradation, prolonged hospital stay, increased risk of complications, reduced response to treatment, shorter survival time, reduced quality of life and increased morbidity and mortality [4,21,22]. Moreover, the nutritional status affects the outcome of treatment and the peri- and postoperative course. If oral intake is insufficient and weight loss is present, nutritional support must be considered for patients with pancreatic cancer. Therefore, early nutritional support is a central component to stabilize body weight and composition and quality of life in these patients. Various studies have evaluated the effect of nutritional counseling and nutritional support on weight, body composition and quality of life. These trials showed an improvement in nutritional status and benefits for cancer patients including pancreatic cancer patients [8,23–27]. Pancreatic mass lesions can be a manifestation of either pancreatic cancer or of chronic pancreatitis. A diagnostic distinction is not always possible by outpatient imaging alone [27] and may require extensive inhospital work-up including endoscopic ultrasound (EUS) guided biopsy [28,29]. Diagnostic work-up is a critical situation because patients experience multiple fasting periods that can aggravate the problem of weight loss and malnutrition. However, to the best of our knowledge, no study has addressed the effect of fasting periods before diagnosis of pancreatic or biliary tract cancer on weight loss, body composition and quality of life. The aim of the present study was to evaluate whether an early in-hospital peripheral intravenous nutrition during fasting periods would have a beneficial effect on body weight, body composition and quality of life in patients with biliopancreatic tumors.

## Material and Methods

This prospective randomized intervention study was conducted according to regulations of the state chamber of physicians for Mecklenburg-West Pomerania, Germany, and we received approval by the local ethics committee of the University Medicine Greifswald on May 29^th^ 2012 (No.BB61/12). It did not fall under the regulations of either the legal requirements for testing medical devices nor under the legal requirements for testing medications and thus a registration in a clinical trials registry was not mandatory prior to recruitment of patients (S1 Checklist). Due to this reason and because of funding issues the study was launched as early as possible. After translation of the CRF and all protocols into English (S1 and S2 Protocols) it was registered belated at ClinicalTrials.gov (NCT02670265). Currently there are no ongoing and related trials for this intervention study.

### Patient group and study design

**Fig 1:**
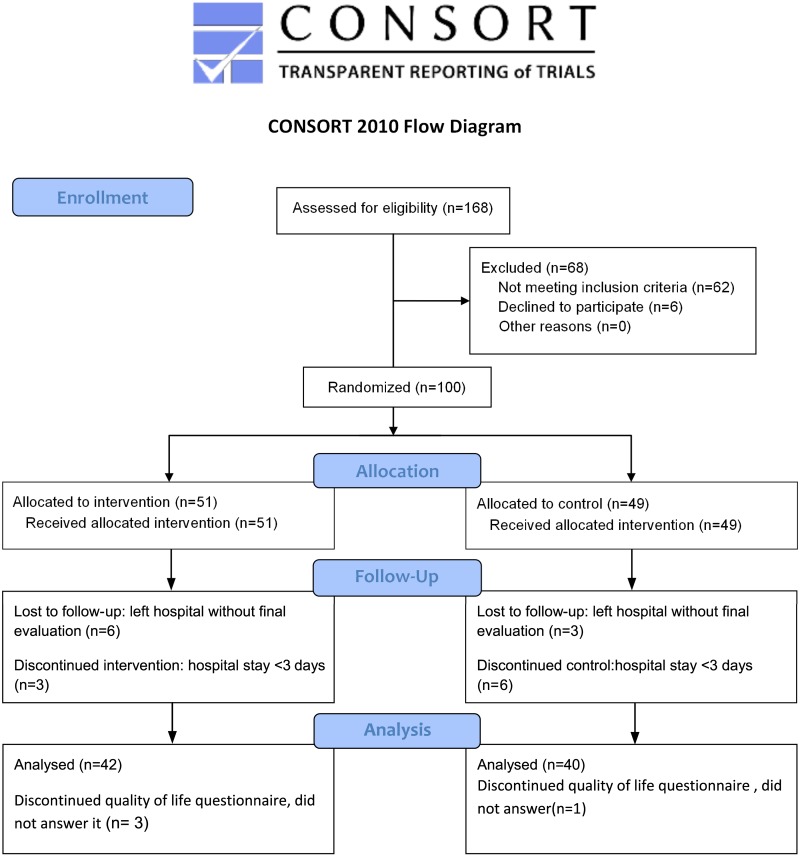
CONSORT diagram of the trial.

Between 18.06.2012 and 11.02.2014 168 patients admitted for a diagnostic work-up of a biliopancreatic mass or lesion at the University Medicine Greifswald (S1 Fig) were screened for this interventional study. All patients gave written informed consent to the study. Patients were considered eligible if they were hospitalized for diagnosis of suspected biliopancreatic mass lesions. The exclusion criteria included the presence of heart- or renal failure (stage III and IV), liver cirrhosis (Child-Pugh B or C), dementia or pregnancy. Patients with a hospital stay of less than three days were excluded due to the short observation time. Of 168 screened patients 100 patients were included in the study and the data of 82 patients were used for the final evaluation (Fig 1) and data of 79 patients could be used for multivariate analysis. 68 patients were not included in the study because 16 patients declined to participate, 46 patients already had a diagnosis (admission for follow-up diagnostics), two patients had a renal failure stage IV and four patients already received parenteral supplementation.

Patients were randomized to either intervention group (1000 ml peripheral intravenous nutrition, 700 kcal) or control group (1000 ml isotonic electrolyte solution). To achieve equal distribution of men and women two block-randomization lists were prepared. Randomization was performed by an independent scientist, who informed the physicians and nutritionist about the group and treatment assigned. These informed the patients and carried out treatment. The study was carried out in an unblinded way. All data were collected at the Department of Medicine A, University Medicine Greifswald.

All measurements (anthropometry, bioelectrical impedance analysis, quality of life) were performed on admission and discharge. Sample size calculation was based on a study of Hasenberg et al. which shows a weight loss of 7 kg ± 2 kg in three months in cancer patients, breaking it down to an average weight loss of 0.6 kg per week, and resulted in a recruitment goal of 48 patients (24 per group) for a statistical power of 95% with an error probability of < 5% [30]. A planned blinded interim analysis after recruitment of 50% of patients showed a significant higher standard deviation for our primary endpoint weight gain than expected from the study by Hasenberg et al and sample size was adjusted accordingly to 50 patients per group to also account for dropouts.

### Anthropometric measurements and bioelectrical impedance analysis

Patients were asked to recall their weight loss over three months before the hospital stay and the duration of weight loss. Actual weight was determined to the nearest of 0.1 kg using a digital scale (Seca 635, Hamburg Germany). Body height was measured to the nearest of 1.0 cm with scale of length (Soehnle professional 5003, Hamburg, Germany). Body mass index (BMI) was calculated as weight (kg) divided by height (m) squared (kg/m²). Nutritional status was assessed using Nutritional Risk Screening (NRS)—2002 [31].

Body composition was determined using a multiple-frequency Bioelectrical Body-Composition Analyzer (BIA 2000 M, Data Input, Darmstadt, Germany). Previous studies have proven validity of bioelectrical impedance analysis (BIA) in assessing of body composition [32,33]. Resistance and capacitance were directly measured with alternating current by 800 mA at 50 kHz and 5 kHz. Data were processed with the software package NutriPlus© (Data Input, Darmstadt, Germany).

### Quality of Life

Quality of life was measured using the *European Organization for Research and Treatment of Cancer Quality of Life Questionnaire* (EORTC-QLQC30). This questionnaire includes five functional scales that interrogate physical, role, cognitive, emotional and social disturbance. Furthermore, it includes six single symptom items. The evaluation was conducted strictly in accordance to the EORTC-QLQC30 scoring manual [34].

### Intervention and oral food intake

1 L Olimel peri 2.5%^®^ (Baxter Germany GmbH Medication Delivery, Unterschleißheim, Germany containing 700 kcal, 25.3 g protein, 30.0 g fat and 75.0 g glucose) was used on fasting days. Parenteral nutrition was administered by a peripheral-venous route. In addition, the intervention group received an adapted supplementation of vitamins (Cernevit^®^, Baxter) and trace elements (Inzolen^®^ Köhler Pharma GmbH, Alsbach-Haehnlein, Germany). 1 L of isotonic fluid (E153, Berlin-Chemie AG, Berlin, Germany) was administered to the control group routinely. This represents a balanced electrolyte solution for intravenous infusion treatment of isotonic and hypotonic fluid losses.

Furthermore, every patient was asked to keep a food diary during hospital stay. A nutritionist instructed the patients how to record their food and oral supplementation. The food diaries were analyzed with the software package OptiDiet^®^ (GOE GmbH, Linden, Germany).

### Statistical Analysis

**Fig 2:**
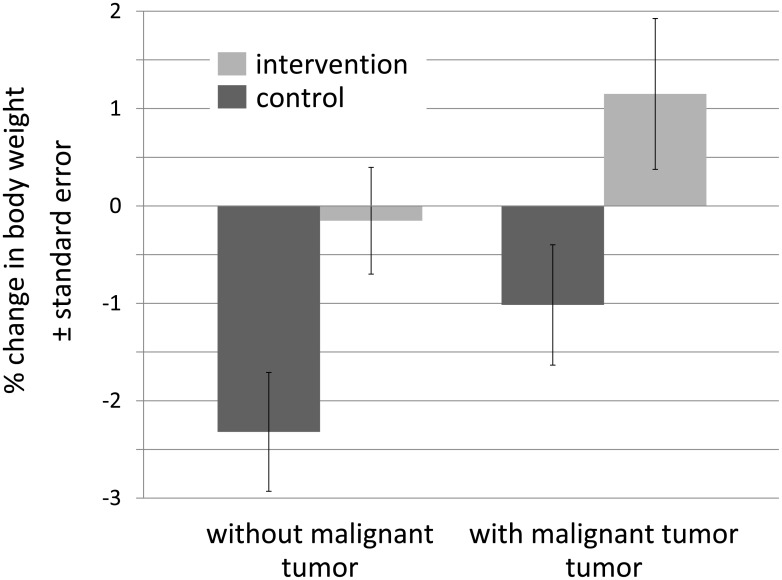
Mean weight changes by intervention and tumor status. Shown are expected marginal means at means of the covariates (sex, age, length of hospital stay, electrolyte solution, weight at admission, malignant tumor, oral supplements and proportion of fasting time). However, the difference between tumor group (total n = 39; control = 19, intervention n = 20) and non-tumor group (total n = 40; control n = 21; intervention = 19) in body-weight change was not statistically significant (p = 0.058).

**Fig 3:**
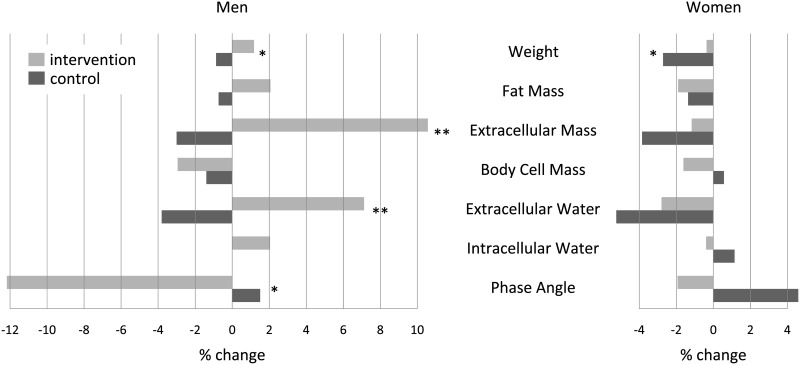
Mean changes in percent from means at admission for intervention and control group in men and women. Shown are expected marginal means at means of the covariates (sex, age, length of hospital stay, electrolyte solution, weight at admission, malignant tumor, oral supplements and proportion of fasting time). Asterisks refer to the statistical significance of the difference between intervention and control group, n = 79. * p < 0.05, ** p < 0.01.

Statistical analyses were performed with Stata 13 (Stata Corp., College Station, TX, USA) by an independent statistician who was neither involved in randomization nor in patient care. Regression analyses were performed to evaluate associations between intervention and changes in weight and body composition. We built one single model for each outcome (difference in weight, fat mass, extracellular mass, body cell mass, phase angle, extracellular water and intracellular water, respectively). All models were adjusted for sex, age, length of hospital stay, dispensation of electrolyte solution, weight at admission, presence of malignant tumor disease, oral supplements and proportion of fasting time. Our modelling approach was as follows: For each outcome we tested if there was 1) evidence of any non-linear relationship and 2) evidence of a sex-specific effect of the intervention. Therefore we used the procedure 'MFPIGEN' in Stata [35] to identify relevant statistical interactions on a multiplicative scale. If the p-value of the interaction term was below 0.4, we chose a model with an interaction term between sex and intervention; otherwise we did not include an interaction term. From these models we reported the adjusted effects of the intervention on changes of each outcome (Table 4). Furthermore, marginal means were displayed in Figs 2 and 3. These are the predicted outcomes derived from the same models whose results are displayed in Table 4. Marginal means were calculated at arithmetic means of all covariates (sex, age, length of hospital stay, electrolyte solution, weight at admission, malignant tumor, oral supplements and proportion of fasting time). All models were run with robust standard errors. Statistical significance was defined as p < 0.05.

## Results

Of 168 screened patients 100 patients were ultimately enrolled in this trial and data from 82 patients were used for final evaluation. Six patients left the hospital before reaching a minimum hospital stay of 3 days and six patients were discharged before a final evaluation could be carried out. Baseline characteristics of the control and intervention group are given in Table 1. The mean age of these patients was 64.9 years with a median stay of seven days with three fasting days. Baseline data obtained from BIA at hospital admission are shown in Table 2, separated by intervention and gender. In women fat mass differed significantly between both groups whereas in men no significant difference was found. All other variables did not show statistically significant differences between both groups at baseline. Patients already reported a mean weight loss of 4.0 kg during the last 3 months before hospital admission (Table 1).

**Table 1:** Baseline characteristics classified in control and intervention and all patients. Data are given as numbers (%) or median (25^th^; 75^th^ percentile), respectively.

	Control (n = 49)	Intervention (n = 51)	p-value	All (n = 100)
Female	20 (40.8%)	23 (45.1%)		43 (43%)
Male	29 (59.2%)	28 (54.9%)		57 (57%)
Age [years]	61.5 (55.6; 71.3)	69.5 (58.2; 75.8)	0.1821	64.9 (56.1; 74.9)
Height [m]	1.72 (1.65; 1.78)	1.72 (1.63; 1.77)	0.5434	1.72 (1.64; 1.78)
Weight at admission [kg]	75.6 (65.0; 85.0)	80.6 (69.8; 87.8)	0.1112	77.0 (67.2; 86.0)
Weight loss before admission (self-reported over last 3 months) [kg]	5.0 (8.2; 0.0)	3.0 (12.0; 0.0)	0.7905	4.0 (10.0; 0.0)
Body Mass Index [kg/m^2^]	25.3 (22.4; 27.8)	26.6 (24.3; 32.0)^a^	0.0298	25.6 (23.2; 29.9)
NRS-2002	2 (1;3)	2 (1;3)	0.8903	2 (1;3)

Data are presented as median with 25*th and 75*th* percentile. Wilcoxon-rank-sum test n = 100. NRS, Nutritional Risk Screening

^a^ Intervention patients are statistically different from control patients, p <0.05.

**Table 2:** Baseline characteristics of body composition classified by intervention and gender.

	Male			Female		
	Control (n = 28)	Intervention (n = 25)	p-value	Control (n = 20)	Intervention (n = 21)) (n = 21)	p-value
Fat Mass [kg]	15.5 (11.2; 22.9)	17.9 (12.9; 23.5)	0.3778	17.1 (13.8; 24.7)	25.8 (24.6; 30.5)	0.0043
Fat Mass [%]	19.1 (15.2; 25.1)	22.5 (18.2; 27.5)	0.2058	27.6 (23.0; 33.6)	35.6 (33.0; 37.5)	0.0102
Total Body Water [l]	46.6 (43.9; 51.4)	45.3 (43.5; 48.5)	0.4542	33.0 (32.0; 34.6)	35.9 (32.0; 38.4)	0.2011
Fat Free Mass [kg]	63.7 (60.0; 70.2)	61.2 (58.1; 66.1)	0.2155	45.1(43.8; 47.3)	49.1 (43.7; 52.5)	0.2011
Extracellular Mass [kg]	34.6 (28.9; 37.0)	31.6 (27.7; 36.9)	0.4382	24.8 (23.3; 26.7)	26.0 (24.0; 27.9)	0.1965
Body Cell Mass [kg]	29.9 (28.1; 35.9)	31.0 (26.2; 33.9)	0.4926	21.5 (18.7; 22.9)	22.0 (19.6; 24.8)	0.1708
ECM/BCM	1.04 (0.91; 1.35)	1.04 (0.95; 1.18)	0.9858	1.16 (1.01; 1.38)	1.13 (1.06; 1.36)	0.9688
Phase angle α [°]	5.4 (4.4; 6.1)	5.4 (4.9; 5.8)	0.9431	4.9 (4.3; 5.6)	5.1 (4.3; 5.3)	0.9063
Extracellular Water [l]	20.2 (18.2; 22.9)	18.9 (17.1; 20.5)	0.2118	12.8 (11.9; 14.0)	14.6 (12.2; 17.1)	0.0924
Intracelluar Water [l]	27.1 (25.3; 29.3)	26.3 (25.1; 27.8)	0.3170	20.3 (19.9; 20.6)	21.0 (19.7; 21.5)	0.2671
REE [kcal/d]	1560 (1500; 1750)	1600 (1450; 1690)	0.4924	1295 (1205;1350)	1310 (1240; 1400)	0.1662

Data are presented as median with 25*th and 75*th* percentile, Wilcoxon rank-sum-test, p <0.05.

ECM, Extracellular Mass; BCM, Body Cell Mass; REE, Resting Energy Expenditure. n = 94.

### Effect of nutritional intervention—Univariate analysis

Nutritional intervention didn’t show a significant increase of body weight compared to electrolyte infusion in the control group (78.4 kg vs. 74.2 kg, p = 0.1812) but showed improvement in BMI (25.8 kg/m^2^ vs. 25.2 kg/m^2^; p = 0.0298). The length of hospital stay was longer in the intervention group (6.0 days vs. 7.0 days, p = 0.0265) (Table 3). The daily oral energy intake during non-fasting in-hospital days was the same in controls and intervention group (1049 kcal vs.1082 kcal). The median of supplementary parenteral nutrition in the intervention group was 1400 kcal during hospital stay. At discharge, about half of the included patients were diagnosed with a malignant tumor of the pancreas or the distal bile duct. Other diagnoses were chronic pancreatitis (n = 7), pancreatic cystic lesion (n = 24), bile duct stones (n = 5) and other GI-tumors (n = 4). In 9 patients eventually no sign of tumor or stone was found.

**Table 3:** Characteristics for control and intervention group.

	Control (n = 40)	Intervention (n = 42)	Difference between admission and discharge		p-value
			Control	Intervention	
Weight [kg]	74.2 (64.2; 84.4)	78.4 (67.5; 87.2)	-0.6 (-1.7; 0.1)	-0.2 (-1.4; 0.5)	0.2172
Body Mass Index [kg/m^2^]	25.2 (21.9; 28.4)	25.8 (23.9; 30.3)	-0.1 (-0.4; 0.0)	0.0 (-0.4; 0.2)	0.3182
Nutritional Risk Screening	2.0 (1.0; 4.0)	2.0 (1.0; 4.0)			0.9800
Length of hospital stay [days]	6.0 (3.0; 8.0)	7.0 (4.0; 11.0)			0.0265
Fasting period [days]	3.0 (2.0; 4.0)	3.0 (2.0; 4.0)			
Parenteral Nutrition [kcal]	0	1400 (700; 2100)			
Diagnosis malignant Tumor	24 (49.0%)	25 (49.0%)			

Data are presented as median with 25*th and 75*th* percentile, Wilcoxon-rank-sum test, p<0.05.

### Effect of nutritional intervention—Multivariate analysis

Regression analyses were performed to evaluate associations between changes in body weight and predictors as well as changes in body composition and predictors. In model 1, which was adjusted by initial weight, gender, hospital stay, dispensation of electrolyte infusion, oral supplements, presence of a malignant tumor and the proportion of fasting days in relation to all in-hospital days a significant association was found. Patients in the intervention group improved weight with an average of 1.7 kg (p = 0.027) during hospitalization compared to the control group. Multivariate analysis also showed a significant increase of extracellular mass (ECM; 4.6 kg; p = 0.021) and extracellular water (ECW; 2.2 l; p = 0.005) in male patients of the intervention group and a decrease of the phase angle by an average of 0.72° (p = 0.038). In females no significant associations between the intervention and the parameters of the bioelectrical impedance analysis were observed (Table 4). Due to adjustment of confounding variables, such as sex and age, the effect of the intervention is much more pronounced in the model (1.7 kg difference of body weight between intervention and control group) compared to the raw data (0.4 kg less weight gain in the intervention group compared to the control group).

**Table 4:** Results of regression analyses: Effects of the intervention on seven different outcomes during hospital stay (weight, fat mass, extracellular mass, body cell mass, phase angle, extracellular water and intracellular water).

	β	p-value	95% confidence interval	
Weight [kg]	1.707	0.027	0.204	3.210
Fat Mass^1^ [kg]—men	0.524	0.229	-0.338	1.387
Fat Mass^1^ [kg]—women	-0.129	0.820	-1.257	0.998
Extracellular Mass^1^ [kg]—men	4.593	0.021	0.714	8.365
Extracellular Mass^1^ [kg]—women	0.686	0.729	-3.254	4.628
Body Cell Mass [kg]	-0.475	0.412	-1.624	0.674
Phase Angle^1^ [°]—men	-0.723	0.038	-1.403	-0.043
Phase Angle^1^ [°]—women	-0.321	0.202	-0.819	0.177
Extracellular Water^1^^,^^2^ [l]—men	2.182	0.005	0.700	3.664
Extracellular Water^1^^,^^2^ [l]—women	0.347	0.649	-1.171	1.864
Intracellular Water^1^^,^^2^ [l]—men	0.555	0.121	-0.152	1.263
Intracellular Water^1^^,^^2^ [l]—women	-0.315	0.414	-1.082	0.452

All models were adjusted for sex, age, length of hospital stay, dispensation of electrolyte solution, weight at admission, tumor, oral supplements and proportion fasting time.

^1^ multiplicative interaction term sex*intervention included; coefficient is the linear combination of main effect and interaction effect.

^2^ length of hospital stay was non-linearly associated with the outcome and thus transformed with the exponent -2.

n = 79.

Patients with a cancer diagnosis experienced a weight gain by 1.15% when randomized to the intervention group (Fig 2). However, the difference was not statistically significant (p = 0.058).

For some outcome variables there was evidence for sex-specific differences in the effect of the intervention; for these variables we added an interaction term for gender. Significant sex-specific changes were found for extracellular mass (ECM), showing a gain of 4.6 kg (p = 0.021) in men, phase angle, where men had a loss of 0.7° (p = 0.038) and for extracellular water (ECW) with a gain of 2.2 l (p = 0.005) in men. Changes in weight and body cell mass (BCM) were equal between men and women (Fig 3).

Only 78 patients out of 100 returned the EORTC-QLQC30 at the end of hospital stay. Regarding quality of life, no significant changes between the intervention and control group were found (Table 5). Points for all other symptoms decreased in their intensity in both groups and both constipation and nausea appeared to decline more in the intervention group (Table 5).

**Table 5:** Changes in Quality of Life parameters during hospital stay.

Characteristic	Baseline		Discharge		Changes in Quality of Life		
	Control	Intervention	Control	Intervention	Control	Intervention	p-value
	n = 49	n = 51	n = 39	n = 39	n = 39	n = 39	
Global Health	58.2 ± 19.7	58.6 ± 19.9	60.8 ± 25.2	62.6 ± 19.9	5.4 ± 25.6	3.1 ± 16.1	0.221
Physical	93.7 ± 13.4	88.6 ± 18.1	93.5 ± 15.3	87.4 ± 19.5	0.7 ± 9.2	-0.7 ± 11.1	0.114
Role	93.8 ± 16.2	90.8 ± 18.9	95.7 ± 15.6	90.6 ± 18.6	2.6 ± 9.0	-0.9 ± 12.1	0.332
Emotional	72.1 ± 23.7	76.5 ± 19.8	74.1 ± 20.2	76.7 ± 23.5	3.6 ± 30.6	-1.0 ± 22.5	0.586
Cognitive	86.6 ± 22.8	87.1 ± 20.1	89.6 ± 22.1	90.6 ± 17.0	3.8 ± 31.7	3.2 ± 18.3	0.945
Social	88.0 ± 23.5	87.1 ± 22.7	90.0 ± 23.1	85.0 ± 27.5	3.6 ± 31.0	-0.3 ± 26.9	0.273
**Symptoms**							
Insomnia	29.9 ± 33.5	40.5 ± 41.8	39.3 ± 38.9	42.7 ± 35.8	8.5 ± 31.3	2.6 ± 40.7	0.340
Appetite Loss	27.9 ± 38.1	26.7 ± 35.0	19.7 ± 30.3	22.2 ± 28.0	-14.5 ± 37.3	-9.4 ± 30.5	0.978
Dyspnea	10.2 ± 21.7	11.8 ± 23.9	2.6 ±16.0	6.8 ± 19.0	-5.1 ± 21.0	-3.4 ± 21.4	0.454
Constipation	9.5 ± 21.5	14.4 ± 30.0	7.7 ± 20.9	10.3 ± 23.1	-0.9 ± 20.9	-4.3 ± 29.8	0.902
Diarrhea	17.0 ± 32.7	11.8 ± 23.9	5.3 ± 16.5	6.8 ± 17.4	-16.7 ± 36.1	-4.3 ± 50.5	0.149
Financial	4.1 ± 14.6	3.9 ± 17.2	6.0 ± 18.5	4.3 ± 17.4	2.6 ± 16.0	1.8 ± 10.8	0.992
Fatigue	34.2 ± 29.8	32.0 ± 26.5	23.9 ± 26.4	24.8 ± 26.7	-13.1 ± 28.4	-7.1 ± 28.2	0.382
Nausea	8.5 ± 19.0	7.5 ± 14.6	5.6 ± 12.3	2.1 ± 7.8	-5.1 ± 23.6	-5.6 ± 12.9	0.412
Pain	24.5 ± 27.0	29.1 ± 32.3	15.8 ± 22.9	21.4 ± 26.8	-10.3 ± 26.9	-5.1 ± 32.3	0.595

Values are presented as mean ± standard deviation. Wilcoxon-rank-sum-test QoL scores range from 0 to 100 and have no units.

### Reported complications of parenteral nutritional support

Most frequently reported adverse events were hyperglycemia and hypertension that were observed in both the intervention and the control group. One case of thrombophlebitis was recorded in the intervention group and one patient of the control group had hypokalemia that needed substitution (S1 Table).

## Discussion

As for other interventional and surgical procedures 3 days of fasting are inevitable when computer tomography scans with oral contrast, EUS or ERCP is performed during in-hospital setting. These fasting days hold a high risk for deterioration due to malnutrition. Nutritional supplementation is an established option to halt further weight loss and cachexia in hospitalized patients. Our results show that parenteral supplementation is indeed able to stabilize body weight in patients who underwent fasting periods during diagnostic work-up, irrespective of present nutritional status. Our results go beyond current ESPEN guidelines that recommend support when inadequate oral food intake is anticipated for more than 10 days [36]. They support the ESPEN recommendation for surgical patients that a combination of enteral and parenteral nutrition should be considered in patients in whom daily energy requirements cannot be fulfilled via the enteral route [37]. Several studies have shown benefits of nutritional supplementation on body weight, body composition and quality of life in cancer patients [25,30,38,39]. However, to the best of our knowledge, this is the first study that investigates the effects of parenteral nutrition during fasting periods for in-patients who would not otherwise require parenteral nutrition because their oral food intake is not impaired. Heterogeneity of our study population with regard to sex, age, length of hospital stay, electrolyte solution, weight at admission, oral supplements taken during hospital stay and proportion fasting time made a multivariate analysis necessary.

Our multivariate analyses showed a statistically significant difference between intervention and control group with regard to body weight. A previous study from Hasenberg et al. investigating parenteral nutritional support in 82 patients with advanced colorectal cancer receiving a palliative chemotherapy showed a benefit for weight, body composition and quality of life (QoL) [30]. There, mean weight loss in the last three months before enrollment was 7.0 kg, whereas our patients reported an average loss of 4.0 kg for the same period. In contrast to the aforementioned study [30] that included only patients with cancer in advanced stage, we included all patients with suspected tumors of the pancreas or distal bile duct. Parenteral nutrition stabilized BMI, in addition BCM, ECM and total body water remained stable, whereas we have noticed a loss of BCM and an increase of total body water and ECM in our study, representing a deterioration of nutritional status. A shift of the ECM/BCM index towards ECM indicates a risk for protein-energy malnutrition because a healthy person has a greater BCM than ECM [32]. Wallengren and co-workers found a higher loss of muscle mass in men with advanced tumor stages than in women. Furthermore, this loss was associated with age, tumor type and concomitant inflammatory conditions [19]. In addition, a decrease of phase angle was seen in our patients that reached significance in male patients. A declining phase angle reflects lower cell membrane integrity and cell function [40].

Our observation period corresponded exactly to the length of hospital stay (median seven days) and therefore was clearly shorter than the observation time in the study of Hasenberg et al. (60 weeks).

Other studies showed an improvement in body composition assessed by BIA. In patients with advanced pancreatic cancer receiving parenteral nutrition an improvement of the ECM/BCM index was observed [23]. However, duration of therapy was much longer than in our study and the authors did not focus on fasting periods during medical examinations. In addition, interpretation of BIA results should be done with caution in individuals with abnormal hydration status, e.g. patients with advanced tumor diseases [33].

Some studies have concentrated on weight stabilization and its association with QoL [8,38,39,41,42]. Patients with unresectable cancer who showed a stable weight (≤1 kg weight loss) after an eight—week nutrition intervention improved in QoL and even showed prolonged survival [8]. Again treatment period was considerably longer than in our study that was only restricted to the in-patient time that was approximately one week. These results also indicate that a nutrition therapy should be initiated as soon as possible. Uster et al., who examined cancer outpatients for effects of nutritional therapy, reported a higher energy and protein intake but no benefit on QoL in their intervention group. Surprisingly, the standard group had a significantly better QoL [41]. In our study physical, role, emotional and social function developed divergently between parenterally fed patients and the controls during hospital stay but were not significant, thus representing only a trend. There are several reasons for a reduced QoL at the end of the observation period. First, QoL decreases when patients are confronted with the diagnosis of a malignant disease. Second, diagnostic procedures are burdened with high physical and mental stress on patients, which further affects QoL. Third, patients might assume that parenteral nutrition implies a more serious disease and this factor can also influence QoL.

Most of studies have observed patients over a longer time period for assessment of QoL. Vashi et al. investigated advanced cancer patients, who received home parenteral nutrition and found an improvement in QoL, nutritional status and functional status. The greatest benefit was found in patients, who received home parenteral nutrition for over 3 months [39].

Data on effects of parenteral nutrition in benign diseases are sparse. Parenteral nutrition of malnourished patients with chronic bowel diseases for four weeks improved BIA results, physical activity and quality of life [43]. The authors recommended an early initiation of nutritional intervention. Furthermore, a meta-analysis of 17 studies showed evidence that nutritional supplementation for at least two weeks in patients with chronic obstructive pulmonary disease (COPD) improved body weight, respiratory muscle strength and quality of life. Effects were most distinct in malnourished people [44].

We encountered divergent results on QoL in our study and did not find significant changes in the answers to the EORTC-QLQC30 questionnaires. There was some evidence that clinical symptoms such as constipation or nausea might improve over short periods. Notably, these effects occur irrespective of the presence of malnutrition as we also enrolled patients without clinically overt malnutrition. These findings suggest that the indication for supplemental nutrition and nutritional counseling should be made liberally in patients with suspected cancer diagnosis.

There are limitations of our study that should be taken into consideration. First, parenteral supplementation was not individualized to body weight due to the standardized protocol, which permitted greater feasibility in routine clinical practice. Second, both intervention and observation period were rather short since follow-up was restricted to the in-hospital period. Due to a rural catchment area of our university hospital patients would be forced to commute long distances for any follow-up investigation that might have led to high dropout rates. Furthermore, some patients left the hospital without informing staff so that the final interview could not be carried out. When necessary, e.g. in cases of volume depletion, some patients of the intervention group received additional electrolyte solution. This fluid can lead to weight gain of patients, too. Additionally, administered volume can also explain the increase of extracellular water observed in the intervention group. However, we made sure that the parenteral nutrition was applied as per protocol in these patients. Some patients with biliopancreatic tumors develop biliary obstruction leading to necessity of ERCP and stent insertion. These procedures were not recorded in our study. Acknowledging the fact that removal of biliary obstruction can raise quality of life and body weight therapeutic endoscopic interventions are additional potential confounders.

In conclusion, we were able to demonstrate that parenteral nutritional supplementation has a stabilizing effect on patients’ weight and body composition during in-hospital fasting periods. Particularly, patients with a cancer diagnosis benefited from the nutritional intervention. Future studies with maybe longer periods of parenteral nutrition will be necessary to clarify whether long-term effects such as patient survival, complications or re-admissions will be affected, too. We were not able to detect a significant improvement in QoL due to parenteral nutrition during diagnostic work-up periods although some symptoms improved. In patients with suspected biliopancreatic tumors further weight loss should be prevented by parenteral nutrition as soon as they are admitted to hospital.

## Supporting Information

S1 Checklist CONSORT 2010 checklist. (DOC)

S1 Fig Flow-chart of diagnostic work-up for bilio-pancreatic lesions at University Medicine Greifswald. *Examinations are usually performed during 9 a.m. to 4 p.m. **In case of concomitant biliary stricture and liver metastasis an additional hospital day will be necessary.
(DOCX)

S1 Protocol Study protocol in English. (DOCX)

S2 Protocol Study protocol in German. (DOC)

S1 Table Reported complications in the intervention and control group during hospital stay. (DOCX)

## Abbreviations

BCM: Body Cell Mass
BIA: Bioelectric Impedance Analysis
BMI: Body Mass Index
ECM: Extracellular Mass
ECW: Extracellular Water
EORTC-QlQc30: European Organization for Research and Treatment of Cancer Quality of Life Questionnaire
EUS: Endoscopic Ultrasound
ICW: Intracellular Water
NRS: Nutritional Risk Screening
QoL: Quality of Life
