# Supplementary material for: Early Parenteral Nutrition in Patients with Biliopancreatic Mass Lesions, a Prospective, Randomized Intervention Trial
Source: PLoS One. 2016 Nov 18;11(11):e0166513. doi: 10.1371/journal.pone.0166513 (PMC5115751; doi:10.1371/journal.pone.0166513)
Supplement: S1 Fig — *Examinations are usually performed during 9 a.m. to 4 p.m. **In case of concomitant biliary stricture and liver metastasis an additional hospital day will be necessary. (DOCX) [file pone.0166513.s002.docx]

**S1 Figure: Flow-chart of diagnostic work-up for bilio-pancreatic lesions at University Medicine Greifswald.** ^*^Examinations are usually performed during 9 a.m. to 4 p.m. ^**^In case of concomitant biliary stricture and liver metastasis an additional hospital day will be necessary.
