# Supplementary material for: Early Parenteral Nutrition in Patients with Biliopancreatic Mass Lesions, a Prospective, Randomized Intervention Trial
Source: PLoS One. 2016 Nov 18;11(11):e0166513. doi: 10.1371/journal.pone.0166513 (PMC5115751; doi:10.1371/journal.pone.0166513)
Supplement: S1 Protocol — (DOCX) [file pone.0166513.s003.docx]

**Study Protocol**

**NUPAN Trial**

**Prospective Randomized Intervention Trial for Early Parenteral Nutrition compared to normal standard care in Patients with Biliopancreatic Mass Lesions**

University Medicine Greifswald

Department of Medicine A

Nutritional Medicine

Ferdinand-Sauerbruch-Str

17475 Greifswald

Tel: 03834/86-6690/-7268

Fax: 03834 /867234

Table of Contents

Summary 4

Study group and Responsabilities 5

Design of the Study 7

**Aims** 7

Primary Endpoint 7

Secondary Endpoints 7

Recruitment of the study population 8

**Inclusion and Exclusion criteria** 8

Flowchart 10

Background: 10

Weight loss and sarcopenia in disorders of the pancreas and the bile ducts 10

Diseases of the pancreas and biliary tract 12

BMI (Body mass index) 15

Bioelectric impedance analysis (B.I.A.) 15

L3-computed tomography 15

Magnetic resonance imaging (MRI) 16

Optidiet 16

Measurement of laboratory parameters 16

Nutritional therapy 16

Initiation of the study 17

Study outline 17

Study termination 18

Statistical considerations 18

Ethical and data safety issues 19

Results 20

Literature 21

# Summary

**Title:**

**Prospective Randomized Intervention Trial for Early Parenteral Nutrition compared to normal standard care in Patients with Biliopancreatic Mass Lesions (NUPAN Study)**

Intervention: Parenteral nutrition

Study design: randomized, prospective, controlled interventional study

Patient group: Patients with biliopancreatic mass lesions that were admitted to hospital

Number of patients: 28 (14 individuals in each group)

Interventional group: daily parenteral nutrition on fasting periods during hospital stay

Control group: isotonic electrolyte solution

Endpoints:

Primary: Weight loss during hospital stay

Secondary:

- Assessment of sarcopenia by BIA and CT –scan
- Laboratory results: total blood count, triglycerides, HDL-cholesterol, LDL-cholesterol, albumine, alanine aminotransferase, aspartate aminotransferase, gGT, alkaline phosphatase, creatinine, blood urea nitrogen, bilirubin, TSH, fT_3_, fT_4_
- Analysis of food patterns
- Analysis of body composition (bioelectric impedance analysis: cellular mass, phase angle, ECM, BCM, ratio ECM/BCM Index, quantitative L3-CT)
- Quality of life

# Study group and Responsibilities

***Study group:***

Prof. Dr. M. Lerch (Department of Medicine A, University Medicine Greifswald)

- Principal Investigator -

Dr. M. Kraft (Department of Medicine A, University Medicine Greifswald)

Dr. J.-P. Kühn (Department of Radiology and Neuroradiology)

M.Sc. oec. troph. Janine Krüger (Department of Medicine A, University Medicine Greifswald)

***Physician Investigator:***

Dr. M. Kraft (Department of Medicine A, University Medicine Greifswald)

***Data and Safety Issues:***

Dr. Eckhard Weber (Department of Medicine A, University Medicine Greifswald)

***Statistics:***

Dr. Peter Meffert (Institute for Community Medicine/SHIP-KEF)

***Contact:***

*Prof. Dr. Markus M. Lerch*

Department of Medicine A

University Medicine Greifswald

Ferdinand-Sauerbruch-Str.

17475 Greifswald

Tel.: 03834-867230 Fax: 03834-867234

E-mail: [lerch@uni-greifswald.de](mailto:lerch@uni-greifswald.de)

*Dr. Matthias Kraft*

Department of Medicine A

University Medicine Greifswald

Ferdinand-Sauerbruch-Str.

17475 Greifswald

Tel.: 03834-867236 Fax: 03834-867234

E-mail: [matthias.kraft@uni-greifswald.de](mailto:matthias.kraft@uni-greifswald.de)

Dr. Jens-Peter Kühn

Department of Radiology and Neuroradiology

Ferdinand Sauerbruch Str.

17475 Greifswald

Tel.:03834-8622170

E-Mail: [kuehn@uni-greifswald.de](mailto:kuehn@uni-greifswald.de)

M.Sc. oec. troph. Janine Krüger

Department of Medicine A

University Medicine Greifswald

Ferdinand-Sauerbruch-Str.

17475 Greifswald

Tel.: 03834-866690 Fax: 03834-867234

E-mail: janine.krueger@stud.uni-greifswald.de

Dr. Peter Meffert

Institute for Community Medicine/SHIP-KEF

University Medicine Greifswald

Walther-Rathenau-Straße 48

17475 Greifswald

E-Mail: peter.meffert@uni-greifswald.de

Telefon: +49 3834 86-19579

# Design of the Study

### **Aims**

The aim of the present study is to evaluate whether an early in-hospital peripheral intravenous nutrition during fasting periods would have a beneficial effect on body weight, body composition and quality of life in patients with biliopancreatic tumors compared to standard medical care.

### Primary Endpoint

- Weight loss during hospital stay

### Secondary Endpoints

- Assessment of sarcopenia by BIA and CT –scan
- Laboratory results: total blood count, triglycerides, HDL-cholesterol, LDL-cholesterol, albumine, alanine aminotransferase, aspartate aminotransferase, gGT, alkaline phosphatase, creatinine, blood urea nitrogen, bilirubin, TSH, fT_3_, fT_4_
- Analysis of food patterns
- Analysis of body composition (bioelectric impedance analysis: cellular mass, phase angle, ECM, BCM, ratio ECM/BCM, quantitative L3-CT)
- Quality of life

This is a monocentric, prospective randomized trial directed by the University Medicine Greifswald, Department of Medicine A.

The study population consists of:

**Interventional group:** Patients with biliopancreatic tumors who receive parenteral nutrition during in-hospital diagnostic work-up.

**Control group:** Patients with biliopancreatic tumors who receive isotonic electrolyte infusion during in-hospital diagnostic work-up.

## Recruitment of the study population

Recruitment was performed by the Department of Medicine A, University Medicine Greifswald. All patients who were admitted for diagnostic workup of an unknown biliopancreatic lesion were screened.

Patients were partly recruited from the PANRAD study, a prospective monocentric observational trial, that compared computed tomography, magnetic resonance imaging (MRI) and endoscopic ultrasound in individuals with an unknown pancreatic lesion or suspected malignancy.

After recruitment and written informed consent patients were randomized in either the interventional or control group. Participants were examined for medical history, nutritional status and quality of life (EORTC QLQ-C30). Routine blood samples were taken at admission. No additional blood samples were required for the study.

Every patient was asked to keep a food diary during hospital stay that was returned when discharged from hospital. A nutritionist instructed the patients how to record their food and oral supplementation. The food diaries were analyzed with the Software OptiDiet^®^ (GOE GmbH, Linden, Germany).

On the day of hospital discharge study participants were examined again for nutritional status (weight, BIA results) and quality of life. Imaging consisted of at least a computed tomography (CT) and magnetic resonance imaging (MRI) that was essential for diagnostic workup. Imaging results were used for sarcopenia assessment and compared to BIA readings.

Parenteral nutrition was given in the interventional group. A *Follow up* was planned after 3 months for re-evaluation of anthropometric parameters and nutritional status.

### **Inclusion and Exclusion criteria**

*Inclusion criteria*

- Patients admitted to hospital for diagnostic workup of a biliopancreatic lesion
- Karnofsky-index > 60

*Exclusion criteria*

- Denial of participation
- Hospital stay < 3 days
- age < 18 years
- Karnofsky-index < 60
- Pregnancy
- Dementia
- Chronic kidney failure (stage III and IV)
- Liver cirrhosis Child Pugh > B
- Heart insufficiency NYHA III - IV

## Flowchart

**Screening of all patients who were admitted for diagnostic work-up of a biliopancreatic lesion**

**Data collected at study inclusion**

- weight
- body composition (BIA)
- food record questionnaire
- Laboratory parameters
- quality of life questionnaire
- L3-CT und MRT, if available
- Laborparameterl

**Randomization**

**Control group**

(1000 ml isotonic electrolyte solution)

**n=17**

**Interventional group**

(1000 ml peripheral intravenous nutrition)

**Data collected at discharge and 3 months later**

- weight
- body composition
- food record questionnaire
- Laboratory parameters
- quality of life questionnaire
- L3-CT und MRT, if available

#

# Background:

## Weight loss and sarcopenia in disorders of the pancreas and the bile ducts

Under- or malnutrition of patients can impose severe problems for therapy (McWhirter J and Pennington C.R.; 1994). In a study by Pierlich et al. in 2006 (Pierlich et al., 2006) 1886 patients (from twelve German and one Austrian hospital) were examined for their nutritional status. 27.4% of all patients had diagnosis of malnutrition. Malnutrition was associated with a lower BMI, further weight loss and a decrease of muscular mass and fatty tissue. Highest prevalence of malnutrition was observed in patients from geriatrics, oncology and gastroenterology.

There are three main risk factors that predispose for malnutrition: 1. age > 70 years, 2. presence of malignancy and 3. intake of more than five drugs (Pirlich et al., 2006).

Almost 90% of patients reported a significant weight loss at time of diagnosis (Sharma et al., 2011). Sometimes weight loss was at least 10% of total body weight. Due to frequent fasting periods during hospital stay and numerous diagnostic examinations patients have a high risk of further weight loss.

Since malnutrition leads to higher morbidity, mortality and a prolonged hospital stay with a concomitant increase of costs in health care, its early diagnosis or prevention is very important (Correia et al., 2003). Malnutrition should be corrected before major surgery because it increases peri- and postoperative morbidity and mortality. Therefore an early correction of malnutrition is an essential part for medical therapy.

Hasenberg et al. (2010) showed that early parenteral nutrition ameliorates quality of life, nutritional status and decreases toxicity of chemotherapy. Patients on parenteral nutrition maintained their BMI (body mass index), BCM (body cell mass) and body fat in contrast to individuals who only received enteral nutrition (Hasenberg et al., 2010).

Body weight loss on the basis of malignant diseases first leads to sarcopenia (loss of muscular tissue) and in later stages to cachexia (Fearon, 2011). Sarcopenia correlates with higher morbidity and mortality and prolongs hospital stay and therefore it should be diagnosed as early as possible (Gray et al., 2011).

Diagnostic tools are bioelectric impedance analysis (BIA), L3-computed tomography (L3-CT) and magnetic resonance imaging (MRI). In routine clinical practice bioelectric impedance analysis is most frequently used. This method is non-invasive, easy to handle and not costly. So far there are no data available showing superiority of any of these methods.

## Diseases of the pancreas and biliary tract

**Physiology of the pancreas**

The pancreas plays an important role for digestion and absorption of nutrients. More than ten different digestive enzymes are secreted (ca. 1-2 l fluid secretion/day). The organ consists of two different parts: the exocrine part, that synthesizes digestive enzymes, i.e. lipase, amylase and peptidases for cleavage of fat, carbohydrates and proteins, and the endocrine part, that produces hormones such as insulin and glucagon. Insufficiency of either the exocrine or the endocrine part affects the patient negatively.

**Disorders of the pancreas**

**Chronic pancreatitis**

Chronic pancreatitis is a chronic inflammation of the pancreas that is characterized by loss of functional tissue that ultimately leads to endocrine or exocrine insufficiency.

**Causes:**

- Chronic alcohol abuse
- Hereditary
- Autoimmune
- Hypercalcemia or hyperparathyroidism
- Pancreatic duct obstruction, ie. due to scars or tumors (Remy Meier, 2006)

Chronic pancreatitis is a known risk factor for pancreatic cancer. Patients with hereditary pancreatitis are at higher risk for pancreatic carcinoma, too. These individuals need to be monitored regularly for early detection of cancer.

**Pancreatic tumours**

Pancreatic tumours can either be of benign or malignant origin. Benign tumours are very rare whereas malignant tumours make up around 90% of all pancreatic lesions (Sharma et al 2011). Pancreatic cancer is the fourth leading cause of death in the U.S. accounting for approximately 227.000 deaths per annum (Vincent et al., 2011). In industrialized countries pancreatic carcinoma is the second most frequent gastrointestinal tumour after colorectal cancer. 5-year survival rate is less than 5%, median survival rate is 5 months.

Histologically, 85% of all carcinomas of the pancreas are ductal adenocarcinomas (PDAC). While 78% of all carcinomas are found in the pancreatic head the remaining 22% are almost equally distributed to the pancreatic body and tail (Sharma et al. 2011).

Cystic pancreatic tumours form a minority of pancreatic neoplasms. There are no specific symptoms for these pancreatic tumours. Abdominal pain, acute pancreatitis by unknown reason, diabetes mellitus, changes of stool consistency (steatorrhea) or jaundice can suspect for a pancreatic tumour.

Cystic pancreatic tumors are subdivided in:

1. serous cystadenomas
2. mucinous cystadenomas
3. cystadenocarciomas
4. intraductal papillary mucinous neoplasias (IPMN)

Serous cystadenomas consist of either one single (oligocystic) or multiple cysts (polycystic). Their pathogenesis is not entirely understood. Mostly, these tumours are benign (97%). Mucinous cystadenomas are almost exclusively found in women and are solitary lesions in the pancreatic tail or body. Although these tumours are mostly benign a malignant transformation is possible. Intraductal papillary mucinous neoplasias (IPMN) are mucin-producing tumours that can develop on a single or multiple locations in the pancreas.

Pathogenesis of pancreatic cancer is only partly understood. One hypothesis is that they arise from ductal cells and transform from pancreatic intraepithelial lesions (PanINs) to invasive carcinoma. Incidence shows a peak between the ages of 50-70 years. Besides hereditary chronic pancreatitis there are exogenous risk factors such as nicotine and alcohol abuse. Smoking is ascribed to be a risk factor for pancreatic cancer in about 20% of cases (Vincent et al., 2011).

Secondary, male sex, diabetes mellitus and obesity are considered to be risk factors for pancreatic cancer. Moreover, history of acute pancreatitis increases the risk of pancreatic cancer by six fold.

Typical symptoms are abdominal pain, sometimes radiating pack pain, jaundice and weight loss (Reissfelder et al., 2007). Weight loss is further increased by presence of anorexia, maldigestion and –absorption. Cancer patients quite often develop tumour cachexia that leads to increased morbidity and mortality (Fearon K.C.H. and Baracos V.E.; 2010).

**Cholangiocellular carcinoma**

Cholangiocellular carcinoma is a malignant tumour of the biliary tract and derives from either the intrahepatic or extrahepatic bile ducts. Tumours located on the confluence of the right and left hepatic bile duct are named Klatskin tumour.

Intrahepatic cholangiocarcinomas are found in 10%, hilary and extrahepatic carcinomas in 25% and 65% of all cases (Lim, 2003). Most often these tumours are adenocarcinomas (Khan et al., 2008; Lim, 2003). Incidence of cholangiocarcinomas is increasing worldwide and is highest in Thailand.

In Western countries risk factors are primary sclerosing cholangitis that increases the risk by up to 30% (Rosen, 1991). In Asia liver fluke Opistorcis viveririne is an additional risk factor. Cholelithiasis, chronic liver diseases (hepatitis B and C), liver cirrhosis and smoking increase the risk for cholangiocarcinoma as well. Moreover, men with age > 70 years are at higher risk for this type of tumour (Khan et al., 2008).

Diagnosis is established by imaging, histopathological and laboratory methods. Similar to pancreatic cancer cachexia also occurs in patients with cholangiocarcinoma that further increases morbidity and mortality.

**Periampullary carcinomas**

About 0.5% of all malignant gastrointestinal tumours are periampullary carcinomas (Gaßler, 2012). These tumours arise in the vicinity of the ampulla of Vater and originate from the pancreas, the duodenum, the distal common bile duct or the papilla (Martin J et al., 2012).

Mean age at diagnosis is between 60 and 70 years. In the last 30 years incidence of periampullar carcinoma was increasing and 5-year survival was between 43-66% (Kohler et al., 2011). Clinical signs are jaundice, diarrhoea, weight loss and fatigue. Other symptoms are abdominal pain, nausea and fever (Martin et al., 2012).

**Methods**

**Evaluation of nutritional status, anthropometry**

Anthropometric measurements are used to assess the composition and nutritional status of the body. Relevant parameters are height [m], body weight [kg] and BMI (Body mass index) [kg/m²].

### BMI (Body mass index)

BMI is defined as the ratio body weight [kg] divided by square body height [m²].

The formula is:

BMI [kg/m²]= body weight [kg]/ body height [m²]

According to BMI patients can be classified as underweight (BMI<18,5kg/m²), normal weight (BMI 18.5 - 24.9kg/m²) or overweight and obese individuals (overweight: BMI >25kg/m² and <30kg/m², obese: BMI> 30kg/m²).

### Bioelectric impedance analysis (B.I.A.)

Bioelectric impedance analysis is used for determination of body composition. It is a widely used and non-invasive method. Technically two electrodes are placed on the patient’s body surface and a weak electric field is be generated that is not felt by the patient. Alternating current is of 800 mA with a frequency of 50 kHz and 5 kHz (Data Input-MBM 2000). There are two types of electric resistances that are determined; one is water resistance (R) out of which body fluid content, lean body mass and fat will be determined. Cellular resistance (Xc) is the second parameter and gives information on organ and muscular mass of the body. Final body composition is calculated by *Nutri Plus software.*

### L3-computed tomography

Computed tomography is done during routine workup for lesions or as a part of the PANRAD trial. No additional CT-scan is necessary for this study. Analysis of data is done by *Osirix* software.

In preparation for the CT scan patients have been fasting over night. After drinking of 500 ml water the patient has to lie on his right side for 5 minutes to allow the water reach the duodenum. Two ampullas of Buscopan® in a dilution of 1:10 are administered intravenously. Biphasic CT-scan is performed using 100 ml Accupaque® or Visipaque® intravenously with infusion speed of 3 ml/sec.

### Magnetic resonance imaging (MRI)

MRI was obtained either as part of the PANRAD trial or from preceding outpatient diagnostic workup. MRI data are used as an alternative method for estimation of body cell mass.

The university medicine Greifswald uses a 1.5 Tesla tomograph (1.5 Tesla Magnetom Avanto, Siemens Health Care, Erlangen, Germany). There is a standardized protocol for the MRI examination that is further outlined in the PANRAD trial. At least 10 minutes are allocated for right positioning of the patient and pre-adjustments. No contrast media is given to the patient.

###

### Optidiet

OptiDiet gives information and recommendations on a variety of diets and allergies and also contains data on nutritional values of nutrients. Optidiet is used for preparation of nutrition protocols and detailed calculation of nutritional components. Recommendations are based on the guidelines of the German Society for Nutrition (Deutsche Gesellschaft für Ernährung, DGE).

### Measurement of laboratory parameters

Total blood count, serum glucose, C-reactive protein, triglycerides, HDL-cholesterol, LDL-cholesterol, albumine, alanine aminotransferase, aspartate aminotransferase, gGT, bilirubin, creatinine, blood urea nitrogen, TSH, fT_3_, fT_4_ and electrolytes are part of standard laboratory workup done by the Institute of Clinical Chemistry of the University Medicine Greifswald.

#### Quality of life

*EORTC QLQ-C30*

Quality of life in cancer patients is assessed by EORTC QLQ-C30, a multidimensional questionnaire. The questionnaire consists of 6 subscales that cover physical fitness as well as 3 subscales and 6 items that ask for physical impairment, social role, cognitive, emotional and social impairment, global quality of life, fatigue, nausea and vomiting, pain and some more symptoms.

## Nutritional therapy

Following determination of the individual energy expenditure the correct nutritional support for each patient is initiated to prevent further weight loss. Resting energy expenditure is calculated by the Harris-Benedict formula that is based on individual's body weight, body height, gender and age.

*Harris Benedict, 1919:*Men:

REE = 66,473 + (13,752 * body weight (in kg)) + (5,003 * (body height in cm)) - (6,755 * age in years).

Women:

REE = 655,096 + (9,5634 * body weight (in kg)) + (1,850 * (body height in cm)) - (4,676 * age in years). (Harris Benedict, 1919)

*Nutritional Risk Screening (NRS)*

Nutritional Risk Screening by Kondrup and coworkers estimates the risk for malnutrition and is based on the severity of the underlying disease (metabolic stress) and nutritional status (BMI, history of weight loss). A score is formed out of these parameters that determines the indication for nutritional support.

*Subject Global Assessment (SGA)*

SGA is an easy to handle and reproducible method for assessment of nutritional status in an in- and outpatient setting. Relevant parameters are weight loss, reduced nutritional intake, loss of subcutaneous fatty tissue, gastrointestinal symptoms and impairment of physical fitness.

# Initiation of the study

After approval of the study by the local ethics committee recruitment could be started. In addition this study will be registered at the database ClinicalTrials.gov (NCT).

#

# Study outline

|  | **Admission** | **Discharge** | **Follow up**  **after 3 months** |
| --- | --- | --- | --- |
| Physical examination | **•** | **•** | **•** |
| Informed consent of patient | **•** |  |  |
| Relevant nutritional parameters | **•** | **•** | **•** |
| Nutritional therapy | **•** | **•** | **•** |
| Bioelectrical impedance analysis | **•** | **•** | **•** |
| Quality of life | **•** | **•** |  |
| Determination energy consumption | **•** | **•** | **•** |
| Blood sampling | **•** | **•** | **•** |
| Food recall | **•** |  | **•** |
| If available:  L3-CT  MRT | **•**  **•** |  | **•**  **•** |

# Study termination

The study will be terminated in presence of the following factors:

1. Patient's wish: the patient can withdraw from the study at any time he/she wants.
2. If the therapy is disadvantageous for the patient.

Any deviation or early termination of the study has to be recorded by a member of the study group. If possible examinations that were originally scheduled at the end of the study should be brought forward. The patient has the right to withdraw from the study at any time. Any adverse event will have to be recorded and the patient observed until clarification of the adverse event.

# Statistical considerations

There is a lack of studies investigating the role of parenteral nutrition on weight stabilization during fasting periods of hospitalized patients.

A positive effect of additional parenteral nutrition on weight stabilization (in patients with colorectal cancer) was described in a study by Hasenberg et al. that was conducted in 2010. Mean weight loss was around 7 kg (± 2 kg) in 3 months. In patients who received parenteral nutrition BMI (Body mass index) and BCM (Body cell mass) remained stable. In addition, body fat content was stable in the intervention group but decreased in the control group. On the other hand body fluid content increased in the controls.

Sample size calculation was based on the study of Hasenberg that assumed a weight difference of 2 kg between the intervention and the control group. This resulted in a recruitment goal of n = 28 participants for a statistical power of 80 % and n = 48 for a statistical power of 95% (figure 1). Assumption of a weight difference of only 1 kg would lead to a sample size of n = 106 (statistical power 80%) and n = 184 (statistical power 95%).

Figure 1: Relation of sample size and power

Statistical analysis was done using STATA 11 software. All participants received descriptive analysis. Comparison of patients with parenteral nutrition and the control group was done using non-parametric Mann-Whitney-U-tests for independent groups. For comparison of two methods (BIA with L3-CT or MRI) a Bland-Altman analysis was done.

#

# Ethical and data safety issues

All generated data were recorded in an anonymized way and were stored on a computer in the Department of Medicine A, Section for Nutritional Medicine. Imaging files were kept in an anonymized way as well but remained in the PACS-database of the Department of Radiology and Neuroradiology.

Data were saved electronically in a pseudonymized way using an identification code. All data were stored on one computer belonging to the Department of Medicine A, University Medicine Greifswald. Only members of the study group were authorized to have access to pseudonymized data.

#

# Results

Patients who are admitted to hospital for diagnostic workup of biliopancreatic lesions are often exposed to involuntary fasting periods. During that time weight loss can occur. Parenteral nutritional supplementation is intended to avoid/reduce weight loss and to improve nutritional status in presence of malnutrition.

The results from the study of Hasenberg et al. in 2010 prompted us to hypothesize that a parenteral nutritional supplementation would have beneficial effects for patients. Patients would maintain their weight, body composition (body fat, BCM, total body fluid, cellular components, phase angle) and improve their outcome.

Hasenberg and co-workers (2010) showed that patients with colorectal cancer profited from parenteral nutrition. These patients could keep their weight/BMI on a constant level for longer time. Weight loss was approximately 7kg (± 2 kg) in three months. Since average hospital stay for patients undergoing in-hospital diagnostic examinations is around one week we assume a weight loss of 1-2 kg during that time, unless no parenteral nutrition is given.

This study shall also clarify, whether parenteral nutritional supplementation instead of only electrolyte infusion during hospital stay increases quality of life.

Moreover, we want to compare the results of bioelectric impedance analysis with L3-CT regarding body cell composition. Since computed tomography is believed to be a good tool for estimation of body cell mass we assume that bioelectric impedance analysis will probably be an inferior method.

# Literature

Correia M.I.; Waitzberg D.L.: *The impact of malnutrition on morbidity, mortality, length of hospital stay and costs evaluated through a multivariate model analysis*. Clinical Nutrition, **2003**. 22(3): 235-239

Fearon K.C.H., Baracos V.E.: Cachexia in pancreatic cancer: new treatment options and measures of success. International Hepato-Pancreato-Biliary Association, **2010**.12, 323-324

Fearon K.C.H.: Cancer cachexia and Fat-Muscle Physiology. The New England Journal of Medicine **2011**. 365; 6;565-567

Gaßler N, Knüchel R.**:**Springer-Verlag (2012) **DOI:** 10.1007/s00292-011-1546-8

Grant J.P: *Nutritional Support in Acute and Chronic Pancreatitis*. Surgical Clinical North America 91 **2011** 805-820

Gray C., MacGillivray T.J., Eeley C., Stephens N.A., Beggs I., Fearon K.C.and Greig C.A.: *Magnetic resonance imaging with k-means clustering objectively measures whole muscle volume compartments in sarcopenia/cancer cachexia*. Clinical Nutrition **2011**. 30:106-111

Harris JA, Benedict FG. *A Biometric Study of Basal Metabolism in Man*.

Washington, DC: Carnegie Institute; **1919**. Publication No. 279.

Hasenberg T.; Essenbreis M.; Herold A.; Post S. and Shang E.: *Early supplementation of parenteral nutrition is capable of improving quality of life, chemotherapy-related toxicity and body composition in patients with advanced colorectal carcinoma undergoing palliative treatment: results from a prospective, randomized clinical trial.* Colorectal disease, **2010**. 12, e190-e199

Khan S.A., Toledano M.B. and Taylor-Robinson D.: *Epidemiology, risk factors, and pathogenesis of cholangiocarcinoma***. 2008**. HPB, 10:77-82

Kohler I., Jacob D., Budzies J., Lehmann A., Weichert W., Schulz S., Neuhaus P.and Röcken C.: *Phenotypic and Genotypic Characterization of Carcinomas of the Papilla of Vater has Prognostic and Putative Therapeutic Implications***.2011** American Journal of Clinical Patholoy.135:202-211

Lim J.H.: Cholangiocarcinoma: *Morphologic Classification According to Growth Pattern and Imaging Findings*. AJR.2003.181:819-827

Martin J.A, Moser A.J, Howell D., Travis A.C. and Savarese: *Ampullary carcinoma: Epidemiology, clinical manifestations, diagnosis and staging.* **2012**

McWhirter J.P. and Pennington C.R.: *Incidence and recognition of malnutrition in hospital*. BMJ **1994**; 308:945-8

Meier R.F.: *Nutrition in pancreatic diseases*. **2006** Best Practice & Research Clinical Gastroenterology. Vol.20, No.3, pp 507-529

Mitsiopoulos N., Baumgartner R.N., Heymsfield S.B., Lyons W., Gallagher D. and Ross R.: Cadaver validation of skeletal muscle measurement by magnetic resonance imaging and computerized tomography. The American Physiological Society, **1998.** 115-122

Pirlich M; Schütz T; Norman K; Gastell S; Lübke H J; Bischoff S C; Bolder U; Frieling T; Güldenzoph H; Hahn K; Jauch K-W; Schindler K; Stein J; Volkert D; Weimann A; Werner H; Wolf C; Zürcher G; Bauer P; Lochs H: *The German hospital malnutrition study*. Clinical nutrition, **2006**.25:563-572

Reissfelder, C.; Koch M., Büchler M.W.; Weitz J.: *Pankreaskarzinom*. Chirurg **2007**. 78:1059-1072

Rosen C., Nagorney D., Wiesner R., Coffey R., La Russo N.: *Cholangiocarcinoma complicating primary sclerosing cholangitis* 1991.Ann Surg 213,1 p. 21-5

Sharma, C.; Eltawil, K.M.; Renfrew Paul D.; Walsh, M.J.; Molinari M.: *Advances in diagnosis, treatment and palliation of pancreatic carcinom*a: *1990-2010.* World Journal of Gastroenterology, **2011** February 21; 21 17(7):867-897

Vincent A; Herman J.; Schulick R.; Hruban R.H.; Goggins M.: *Pancreatic Cancer*. Published online May 26, **2011** www.thelancet.com
