# Supplementary material for: Early Parenteral Nutrition in Patients with Biliopancreatic Mass Lesions, a Prospective, Randomized Intervention Trial
Source: PLoS One. 2016 Nov 18;11(11):e0166513. doi: 10.1371/journal.pone.0166513 (PMC5115751; doi:10.1371/journal.pone.0166513)
Supplement: S2 Protocol — (DOC) [file pone.0166513.s004.doc]

**Studienprotokoll**

**NUPAN-Studie**

**Prospektiv, randomisierte Interventionsstudie zur Stabilisierung des Ernährungsstatus durch frühzeitige parenterale**

**Ernährungssupplementierung im Vergleich zur normalen Standardbehandlung bei Patienten mit Verdacht auf Pankreas- und/oder**

**Gallenwegserkrankungen**

Universitätsmedizin Greifswald

Klinik und Poliklinik für Innere Medizin A

Ernährungsmedizin

Ferdinand-Sauerbruch-Str

17475 Greifswald

Tel: 03834/86-6690/-7268

Fax: 03834 /867234

Inhalt

[Zusammenfassung 4](#__RefHeading___Toc323213412)

[Studienorganisation und Verantwortlichkeiten 5](#__RefHeading___Toc323213413)

[Studiendesign 7](#__RefHeading___Toc323213414)

[Ziele 7](#__RefHeading___Toc323213415)

[Primärer Zielparameter 7](#__RefHeading___Toc323213416)

[Sekundäre Zielparameter 7](#__RefHeading___Toc323213417)

[**Einschluss der Patienten in die Studie** 8](#__RefHeading___Toc323213418)

[Festlegung der Einschluss- und Ausschlusskriterien 9](#__RefHeading___Toc323213419)

[**Flowchart zum Studiendesign** 10](#__RefHeading___Toc323213420)

[Hintergrundinformationen 11](#__RefHeading___Toc323213429)

[**Gewichtsverlust und Sarkopenie bei Pankreas- und Gallenwegserkrankungen** 11](#__RefHeading___Toc323213430)

[**Pankreas-und Gallenwegserkrankungen** 12](#__RefHeading___Toc323213431)

[Methoden 16](#__RefHeading___Toc323213433)

[Ermittlung des Ernährungszustandes, Anthropometrie 16](#__RefHeading___Toc323213434)

[BMI (body mass index) 16](#__RefHeading___Toc323213435)

[Bioelektrische Impedanzanalyse (B.I.A.) 16](#__RefHeading___Toc323213436)

[L3-Computertomographie 16](#__RefHeading___Toc323213437)

[Magnetresonanztomographie 17](#__RefHeading___Toc323213438)

[Optidiet 17](#__RefHeading___Toc323213439)

[Messung der klinisch relevanten Parameter 17](#__RefHeading___Toc323213440)

[Ernährungstherapie 18](#__RefHeading___Toc323213441)

[Studienbeginn 19](#__RefHeading___Toc323213442)

[Studienablauf 19](#__RefHeading___Toc323213443)

[Abbruch der Behandlung 19](#__RefHeading___Toc323213444)

[Statistische Überlegungen 20](#__RefHeading___Toc323213445)

[Ethik und Datenschutz 21](#__RefHeading___Toc323213446)

[Ergebnisse 22](#__RefHeading___Toc323213447)

[Literaturliste 23](#__RefHeading___Toc323213448)

# Zusammenfassung

**Titel:** **Prospektiv, randomisierte Interventionsstudie zur Stabilisierung des Ernährungsstatus durch frühzeitige parenterale Ernährungssupplementierung im Vergleich zur normalen Standardbedingung bei Patienten mit Pankreas- und Gallenwegserkrankungen (NUPAN-Studie)**

Studienintervention: Parenterale Zusatzernährung

Studiendesign: randomisierte, prospektive, kontrollierte Interventionsstudie

Patientenpopulation: Patienten, die zur Abklärung einer fokalen Läsion des Pankreas- oder der Gallenwege (unklare Raumforderung) in die Klinik kommen

Patientenzahl: 28 (14 Personen pro Gruppe)

Interventionsgruppe: routinemäßige parenterale Zusatzernährung 1x täglich im Rahmen des stationären Aufenthaltes während Nüchternphasen im Rahmen von Untersuchungen

Kontrollgruppe: Standard care

Zielparameter:

Primär: Gewichtsverlust während des stationären Aufenthaltes

Sekundär:

- Vergleich BIA mit CT –Daten hinsichtlich Sarkopenie
- laborchemische Parameter: großes Blutbild TG, HDL-Cholesterin, LDL-Cholesterin, Albumin, ALAT, ASAT, GGT, AP, Kreatinin, Harnstoff, Bilirubin, TSH/T_3_/T_4_
- Untersuchung der Ernährungsweise (Optidiet)
- Untersuchung der Körperzusammensetzung (bioelektrische Impedanzanalyse: Zellanteil, Phasenwinkel, ECM, BCM,ECM/BCM Index und L3-CT quantitativ)
- Lebensqualität am Anfang und am Ende des klinischen Aufenthaltes sowie im follow-up

# Studienorganisation und Verantwortlichkeiten

***Studienleiter:***

Prof. Dr. M. Lerch (Klinik für Innere Medizin A, Universität Greifswald)

Dr. med. M. Kraft (Klinik für Innere Medizin A, Universität Greifswald)

Dr. med. J.-P. Kühn (Institut für Diagn. Radiologie und Neuroradiologie)

M. Sc. oec. troph. Janine Krüger (Klinik für Innere Medizin A, Universität Greifswald)

***Prüfarzt:***

Dr. med. M. Kraft (Klinik für Innere Medizin A, Universität Greifswald)

***Unabhängige Daten und Sicherheitsbestimmungen:***

Dr. rer. nat. Eckhard Weber (Klinik für Innere Medizin A, Universität Greifswald)

***Statistiker:***

Dr. Peter Meffert (Institut für Community Medicine/SHIP-KEF)

***Kontaktadressen:***

*Prof. Dr. med. Markus M. Lerch*

Klinik und Poliklinik für Innere Medizin A

Universitätsmedizin, Ernst-Moritz-Arndt Universität Greifswald

Ferdinand-Sauerbruch-Str-

17475 Greifswald

Tel.: 03834-867230 Fax: 03834-867234

E-mail: [lerch@uni-greifswald.de](mailto:lerch@uni-greifswald.de)

*Dr. med. Matthias Kraft*

Klinik und Poliklinik für Innere Medizin A

Universitätsmedizin, Ernst-Moritz-Arndt Universität Greifswald

Ferdinand-Sauerbruch-Str.

17475 Greifswald

Tel.: 03834-867236 Fax: 03834-867234

E-mail: [matthias.kraft@uni-greifswald.de](mailto:matthias.kraft@uni-greifswald.de)

Dr. med. Jens-Peter Kühn

Diagnostische Radiologie und Neuroradiologie

Ferdinand Sauerbruch Str.

17475 Greifswald

Tel.:03834-8622170

E-Mail: [kuehn@uni-greifswald.de](mailto:kuehn@uni-greifswald.de)

M. Sc. oec. troph. Janine Krüger

Klinik und Poliklinik für Innere Medizin A

Universitätsmedizin, Ernst-Moritz-Arndt Universität Greifswald

Ferdinand-Sauerbruch-Str.

17475 Greifswald

Tel.: 03834-866690 Fax: 03834-867234

E-mail: janine.krueger@stud.uni-greifswald.de

Dr. Peter Meffert

Universitätsmedizin Greifswald

Institut für Community Medicine/SHIP-KEF

Walther-Rathenau-Straße 48 • 17475 Greifswald

E-Mail: peter.meffert@uni-greifswald.de

Telefon: +49 3834 86-19579

# Studiendesign

### Ziele:

Ziel dieser Studie ist es, festzustellen, ob Patienten, die zur Abklärung einer Läsion des Pankreas- oder der Gallenwege stationär aufgenommen werden, während des Krankenhausaufenthaltes durch die routinemäßige supplementierende intravenöse Zufuhr von Makro- und Mikronährstoffen in Nüchternphasen einen geringeren Gewichtsverlust aufweisen als Patienten ohne prophylaktische Intervention.

### Primärer Zielparameter

- Gewichtsverlust während des stationären Aufenthaltes

### Sekundäre Zielparameter

- Vergleich BIA mit CT –Daten hinsichtlich Sarkopenie
- laborchemische Parameter: großes Blutbild TG, HDL-Cholesterin, LDL-Cholesterin, Albumin, ALAT, ASAT, GGT, AP, Kreatinin, Harnstoff, TSH/T_3_/T_4_
- Untersuchung der Ernährungsweise (Optidiet)
- Untersuchung der Körperzusammensetzung (bioelektrische Impedanzanalyse: Zellanteil, Phasenwinkel, ECM, BCM,ECM/BCM Index und L3-CT quantitativ)
- Lebensqualität am Anfang und am Ende des klinischen Aufenthaltes sowie im follow-up

Die Durchführung der Studie erfolgt monozentrisch, prospektiv randomisiert (Universitätsmedizin Greifswald, Klinik und Poliklinik für Innere Medizin A sowie Institut für diagnostische Radiologie und Neuroradiolgie).

Die Studienpopulation setzt sich wie folgt zusammen:

**Interventionsgruppe:** Patienten, die zur Abklärung einer unklaren Raumforderung des Pankreas und / oder der Gallenwegen stationär aufgenommen werden, sollen bei Nüchternphasen supportiv **mit** parenteraler Ernährung versorgt werden.

**Kontrollgruppe:** Patienten, die wegen einer unklaren Raumforderung des Pankreas oder der Gallenwege stationär aufgenommen werden und keine routinemäßige Intervention erhalten (standard care).

## Einschluss der Patienten in die Studie

Die Rekrutierung der Patienten erfolgt durch die Klinik und Poliklinik für Innere Medizin A, Universitätsmedizin Greifswald. Es werden Patienten gescreent, welche zur differentialdiagnostischen Abklärung einer unklaren Raumforderung im Bereich des Pankreas oder aber zur weiteren Abklärung eines schmerzlosen Ikterus aufgenommen werden. Diese rekrutieren sich zum Teil aus der bereits laufenden PANRAD Studie, bei der es sich um eine prospektive monozentrische Beobachtungsstudie handelt, in der Patienten mit einer Pankreasläsion unbekannter Dignität und Verdacht auf Malignomen eingeschlossen werden. Verglichen wird in dieser prospektiven Studie die diagnostische Wertigkeit einer Computertomographie, einer Kernspinntomographie (MRT) sowie einer Endosonographie hinsichtlich eines positiven prädiktiven Werts zur Detektion einer Tumorerkrankung des Pankreas.

Nach erfolgter Rekrutierung und schriftlicher Einwilligung der Probanden erfolgt eine Randomisierung in die Kontroll- und Vergleichsgruppe. Am Aufnahmetag erfolgt eine Screeningvisite, in der der Ernährungszustand des Patienten beurteilt wird. Eine Routineblutentnahme wird im Rahmen der stationären Aufnahme durchgeführt. Zusätzliches Blut wird nicht entnommen. Zudem wird ein Lebensqualitätsbogen (EORTC-25, *QLQ-PAN26*) ausgefüllt.

Während des stationären Aufenthaltes soll der Patient ein Ernährungsprotokoll führen. In diesem wird prospektiv die orale Nahrungs- und Trinkzufuhr während des stationären Aufenthaltes protokolliert. Das Ernährungsprotokoll wird am Tag der Entlassung eingeholt und die Menge der während des stationären Aufenthaltes zugeführten Makro- und Mikronährstoffe analysiert (Software Programm Optidiet). Darüber hinaus wird am Entlassungstag erneut der Ernährungszustand (Gewicht, Körperzusammensetzung) bestimmt und der Lebensqualitätsfragebogen ausgefüllt. Im Rahmen der differentialdiagnostischen Abklärung erfolgt zumindest eine Computertomographie, häufig auch zusätzlich eine Magnetresonanztomographie. Die bildgebende Diagnostik wird herangezogen, um eine Quantifizierung der Körpermuskelmasse durchzuführen. Hierbei ist eine Sarkopenie zuverlässig nachweisbar. Diese Daten werden dann mit den BIA –Ergebnissen verglichen.

Die parenterale Ernährungstherapie der Patienten in der Interventionsgruppe wird für jeden Patienten individuell festgelegt. Nach 3 Monaten soll ein *Follow up* durchgeführt werden, indem der Ernährungszustand erneut erhoben wird.

### Festlegung der Einschluss- und Ausschlusskriterien

Einschlusskriterien (Indikationen):

- Patienten, die zur Abklärung einer fokalen Läsion des Pankreas und / oder der

Gallenwege stationär aufgenommen werden

- Karnofsky-Index > 60

Ausschlusskriterien (Kontraindikationen):

- Fehlen der Einverständniserklärung
- Krankenhausverweildauer < 3 Tage
- Alter < 18 Jahre
- Karnofsky-Index < 60
- Schwangerschaft
- Demenzerkrankung
- dialysepflichtige Niereninsuffizienz (Stadium III und IV)
- Leberzirrhose Child Pugh > B
- Herzinsuffizienz NYHA III - IV

## Flowchart zum Studiendesign

**Screening aller stationär neu aufgenommenen Patienten** (Raumforderung des Pankreas oder der Gallenwege)

**Screening des Ernährungszustandes am Tag der Aufnahme**

- Gewicht am Tag der Aufnahme
- BIA-Messung
- Ausgeben Ernährungsprotokoll
- Laborparameter
- QLQ Fragebogen
- L3-CT und MRT wenn vorhanden
- Laborparameterl

**Randomisierung**

**Interventionsgruppe**

(supportive parenterale Ernährung)

**n=17**

**Kontrollgruppe**

(Standard care)

**n=17**

**Screening des Ernährungszustandes am Tag der Entlassung und nach 3 Monaten**

- Gewicht am Tag der Entlassung
- BIA-Messung
- Auswertung Ernährungsprotokoll
- Laborparameter
- QLQ
- L3-CT und MRT wenn vorhanden

# Hintergrundinformationen

## Gewichtsverlust und Sarkopenie bei Pankreas- und Gallenwegserkrankungen

Mangelernährte Patienten stellen in Krankenhäusern ein schwerwiegendes Problem dar (McWhirter J and Pennington C.R.; 1994). Dies zeigte auch die Studie der Gruppe um Pierlich et al. aus dem Jahr 2006 (Pierlich et al., 2006). Untersucht wurden in dieser Studie insgesamt 1886 Patienten hinsichtlich ihres Ernährungszustandes (zwölf Krankenhäuser in Deutschland und eines in Österreich). Dabei wiesen etwa 27,4% der Patienten in diesen Krankenhäusern eine Mangelernährung auf. Die Mangelernährung ging dabei mit einem geringeren BMI, einem unerklärlichen Gewichtsverlust sowie mit einem Muskel- und Fettmasseverlust einher. Die höchste Prävalenz für das Auftreten einer Mangelernährung wurde für folgende Bereiche aufgezeigt: Geriatrie, Onkologie und Gastroenterologie. Es konnten drei Hauptrisikofaktoren identifiziert werden, die zu einer Mangelernährung führen können: 1. Alter >70 Jahre 2. maligne Erkrankungen und 3. die Einnahme von mehr als fünf Medikamenten (Pirlich et al., 2006).

Nahezu 90% der Patienten haben bereits bei Diagnosestellung einen signifikanten Teil ihres Gewichts verloren (Sharma et al., 2011). Nicht selten beträgt dieser Verlust 10% des Körpergewichts. Im Rahmen des stationären Aufenthaltes zum Zwecke der Diagnosestellung kann es bereits zu einem signifikanten Gewichtsverlust kommen, da die Patienten auf Grund der vielen Untersuchungen häufig Nüchternphasen ausgesetzt sind.

Da eine Mangelernährung zu einer erhöhten Morbidität, Mortalität, einem verlängerten Krankenhausaufenthalt und somit auch zu höheren Kosten führen kann, ist es wichtig diese zu verhindern bzw. rechtzeitig zu erkennen (Correia et al., 2003). Insbesondere im Rahmen der Abklärung von malignitäts-suspekten Befunden des oberen GI-Traktes muss mit Blick auf eine zu erwartende chirurgische Intervention betont werden, dass eine präoperative Konditionierung der Patienten bezüglich des Ernährungszustandes hinsichtlich der perioperativen Mortalität und Morbidität essentiell ist und die höchste Evidenz aufweist (Leitlinie der DGEM). Die frühzeitige Verbesserung und das Erhalten des Ernährungszustandes sollte deswegen ein wichtiger Bestandteil der Therapie sein.

Darüber hinaus konnte die Gruppe um Hasenberg et al. (2010) zeigen, dass der frühe Einsatz einer parenteralen Ernährungstherapie die Lebensqualität der Patienten, die Körperzusammensetzung und die Chemotherapie bedingte Toxizität positiv beeinflusst. Bei Patienten mit parenteraler Ernährung blieb der BMI (Body mass index) und die BCM (Body cell mass) konstant im Gegensatz zu den Patienten, die nur orale Zusatzernährung bekamen. Während der Körperfettanteil der Kontrollgruppe innerhalb des Zeitraumes kontinuierlich sank, blieb dieser in der Interventionsgruppe konstant. Bei Patienten in der Kontrollgruppe stieg der Körperwasseranteil (Hasenberg et al., 2010).

Ein Körpergewichtsverlust im Rahmen einer zu Grunde liegenden konsumierenden Erkrankung führt initial häufig zu einer sogenannten Sarkopenie (Muskelabbau), erst spät zeigen sich eigentliche Zeichen einer Kachexie (Fearon, 2011). Da das Ausmaß der Sarkopenie die Lebensqualität maßgeblich beeinflusst, ist es wichtig, diese bei Patienten vorzeitig zu erkennen. Des Weiteren korreliert das Ausmaß der Sarkopenie mit der Mortalität, Morbidität und der Krankenhausverweildauer (Gray et al., 2011). Die Sarkopenie der Patienten kann unter anderem mit Hilfe folgender validierten Methoden ermittelt werden. Bioelektrische Impedanzanalyse (BIA), L3-Computertomographie (L3-CT) und Magnetresonanztomographie (MRT). Im klinischen Alltag wird häufig aus Kosten- und Praktikabilitätsgründen die Bioelektrische Impedanzanalyse (BIA) verwendet, um die Körperzusammensetzung des Patienten zu bestimmen. Im Gegensatz zur CT ist der Patient keinen Röntgenstrahlen ausgesetzt, die Methode ist nicht invasiv, günstiger und ubiquitär verfügbar. Derzeit existieren keine konklusiven Daten hinsichtlich der Überlegenheit der einen oder anderen Methode.

## Pankreas- und Gallenwegserkrankungen

**Physiologie des Pankreas**

Das Pankreas spielt eine wichtige Rolle für die Verdauung und Absorption von Nährstoffen. Ein gesundes Pankreas sezerniert mehr als zehn verschiedene Enzyme zur Verdauung der aufgenommenen Nahrung (ca.1-2l Bauchspeichelsekret pro Tag). Es besteht aus zwei unterschiedlichen Anteilen. Zum einen aus dem exokrinen Anteil, welcher der größte Lieferant von Verdauungsenzymen ist und zum anderen aus dem endokrinen Teil, indem die Hormone Insulin und Glukagon gebildet werden. Zu den Verdauungsenzymen zählen die Lipasen (Fettspaltung), die Amylase (Kohlenhydratspaltung) und die Proteinasen/Peptidasen (Eiweißspaltung). Erkrankungen des Pankreas können zu einer gestörten Funktion führen, welche sich negativ für den Patienten auswirken.

### Erkrankungen des Pankreas

**Die chronische Pankreatitis**

Unter einer chronischen Pankreatitis versteht man eine Entzündung der Bauchspeicheldrüse, die zur Zerstörung des Drüsenapparates führt. Dies äußert sich sowohl in einer fehlenden Absonderung von Verdauungsenzymen als auch in der unzureichenden Produktion von Bauchspeicheldrüsenhormonen wie z.B. Insulin. Dies kann nach mehrjährigem Verlauf zu einer endokrinen bzw. exokrinen Pankreasinsuffizienz führen.

**Ursachen:**

- Chronischer Alkoholkonsum
- Hereditär
- Autoimmune Genese
- Hyperkalzämie und Hyperparathyreodismus
- Chronische Pankreasgangobstruktion durch Narben, Tumore (Remy Meier, 2006)

Das Vorliegen einer chronischen Pankreatitis stellt außerdem einen hohen Risikofaktor für das Entstehen eines Pankreaskarzinoms dar. Besonders Patienten, die an hereditärer Pankreatits leiden, besitzen ein erhöhtes Risiko an einem Pankreaskarzinom zu erkranken. Diese Patienten bedürfen einer regelmäßigen Kontrolle mittels endoskopischer und Bild gebender Verfahren.

**Pankreastumore**

Pankreastumore lassen sich in benigne und maligne Tumore unterteilen, wobei die benignen Tumore sehr selten sind und die malignen ca. 90% der Tumore ausmachen (Sharma et al 2011). Das Pankreaskarzinom ist die vierthäufigste krebsbedingte Todesursache in den USA und führt schätzungsweise zu 227000 Toten pro Jahr (Vincent et al., 2011). In den Industrieländern liegt die Inzidenz an einem Pankreaskarzinom zu erkranken an zweiter Stelle aller gastrointestinalen Erkrankungen hinter dem kolorektalen Karzinom.

Die Überlebensrate 5 Jahre nach Diagnosestellung beträgt weniger als 5% und die mediane Überlebensrate liegt bei 5 Monaten.

Bei fast allen Pankreaskarzinomen handelt es sich um Adenokarzinome, die aus den Epithelien des Pankreasganges entstehen. Diese Karzinome machen etwa 85% aller Pankreaskarzinome aus. Während 78% der Karzinome im Pankreaskopf zu finden sind, verteilen sich die restlichen 22% auf den Pankreascorpus (11%) oder den Pankreasschwanz (11%) (Sharma et al. 2011).

Im Vergleich hierzu stellen die zystischen Pankreastumore prozentual eine Minderheit dar. Wie bei allen Tumoren der Bauchspeicheldrüse gibt es keine spezifischen Symptome. Häufig handelt es sich um einen Zufallsbefund. Oberbauchschmerzen, eine Bauchspeicheldrüsenentzündung, ein plötzlich auftretender Diabetes mellitus, Stuhlveränderungen (Fettstühle) oder eine Gelbfärbung der Augen bzw. der Haut (Ikterus) können aber symptomatisch für einen zystischen Pankreastumor sein. Folgende zystische Tumore werden unterschieden:

a) seröse Zystadenome
b) muzinöse Zystadenome
c) Zystadenokarzinome
d) Intraduktale papilläre muzinöse Neoplasien (IPMNs)

Seröse Zystadenome können sowohl aus einer Zyste (oligozystisch) als auch aus mehreren kleinen Zysten (polyzystisch) bestehen. Der Ursprung und die Entstehung dieser Tumore ist bisher nicht geklärt. In den meisten Fällen handelt es sich um gutartige Tumore (97% der Fälle). Muzinöse Zystadenome haben entwicklungsgeschichtlich ihren Ursprung offenbar aus der Anlage der Keimdrüsen. Diese Tumore findet man fast ausschließlich bei Frauen und sie treten einzeln im Pankreasschwanz oder Pankreaskörper auf. Es handelt sich zumeist um einen gutartigen Tumor (ca. 75% der Fälle), der aber die Fähigkeit besitzt, bösartig zu werden. Intraduktale papilläre muzinöse Neoplasien (IPMNs) sind Schleim produzierende Tumore, die einzeln oder an mehreren Stellen in der Bauchspeicheldrüse auftreten können. Die Ursache für die Entstehung dieser Tumore ist noch immer unklar. Man geht derzeit davon aus, dass sich die Zellen der Bauchspeicheldrüsengänge über verschiedene Stufen langsam verändern, bis hin zu einem bösartigen Tumor, dem Bauchspeicheldrüsenkrebs.

Die meisten Neuerkrankungen treten zwischen dem 50.-70. Lebensjahr auf. Neben der familiären Disposition für chronische Pankreatitis (bei 7-10% der Patienten traten Pankreaserkrankungen bereits in der Familie auf) konnten auch exogene Faktoren nachgewiesen werden, welche das Risiko an einem Pankreaskarzinom zu erkranken, erhöhen. Als erstes ist hier der Nikotin- und Alkoholabusus zu nennen. Etwa 20% der Pankreastumore sind auf einen Nikotinabusus zurückzuführen (Vincent et al., 2011).

Ebenfalls gelten das männliche Geschlecht, Diabetes mellitus und Übergewicht/Adipositas als Risikofaktoren für die Entstehung eines Pankreaskarzinoms. Eine abgelaufene akute Pankreatitis erhöht das statistische Risiko an einem Pankreaskarzinom zu erkranken um das sechsfache.

Typische Symptome, die bei einem Pankreaskarzinom auftreten können, sind Bauchschmerzen, in den Rücken ausstrahlende Schmerzen, Ikterus und Gewichtsverlust (Reissfelder et al., 2007). Der Gewichtsverlust kann durch Anorexie, Maldigestion

und -absorption entstehen. Nicht selten entwickeln die Patienten eine Tumorkachexie. (Fearon K.C.H. and Baracos V.E.; 2010), welche sich in einer gesteigerten Mortalität und Morbidität äußern kann.

**Cholangiozelluläres Karzinom**

Das Cholangiozelluläre Karzinom ist ein bösartiger Tumor der Gallenwege. Man unterscheidet Karzinome, die in den Gallengängen der Leber wachsen (intrahepatisch) von extrahepatischen Gallengangstumoren, die außerhalb der Leber auftreten. Eine Sonderform stellen Tumore dar, die ein hiläres Wachstum aufweisen und als Klatskin-Tumore bezeichnet werden.

Intrahepatische Karzinome machen etwa 10%, hiläre 25% und extrahepatische 65% der gesamten Cholangiokarzinome aus (Lim, 2003). Das Gallengangskarzinom ist ein Tumor der epithelialen Zellen und kommt eher selten vor (Khan et al., 2008; Lim, 2003). Häufig handelt es sich um Adenokarzinome (Lim, 2003). Die höchste Inzidenz an einem Cholangiokarzimon zu erkranken liegt in Thailand vor, allerdings steigt die Erkrankungsrate weltweit.

Zu den Risikofaktoren der Entstehung eines Cholangiokarzinoms zählt in den westlichen Ländern vor allem die primär skleorisierende Cholangitis. Beim Vorliegen einer primär skleorisierenden Cholangitis steigt die Prävalenz auf bis zu ca. 30% (Rosen, 1991).

In Asien zählt der Leberegel Opistorcis viveririne als ein weiterer wichtiger Risikofaktor. Bis zu 10% der Patienten, welche Gallensteine besitzen, können ebenfalls ein Cholangiokarzinom entwickeln. Patienten mit chronischen Lebererkrankungen (Hepatitis B oder C) oder Leberzirrhose haben ebenfalls ein erhöhtes Risiko an einem Cholangiokarzinom zu erkranken. Des Weiteren stellt ein Nikotinabusus einen Risikofaktor dar. Besonders betroffen sind Männer ab dem 70.Lebensjahr (Khan et al., 2008).

Zu den Diagnostikverfahren zählen die Bildgebung, Gewebeuntersuchung und Laboruntersuchungen.

Wie auch beim Pankreaskarzinom kann sich eine Tumorkachexie entwickeln, welche sich auf die Morbidität und Mortalität auswirken kann.

**Karzinom der Papilla vateri**

Etwa 0,5% aller gastrointestinalen malignen Tumoren sind Papillenkarzinome (Gaßler, 2012). Diese Neoplasien entstehen in der Nähe der Papilla Vateri und können dem Pankreas, Duodenum, der Gallengänge oder direkt der Papilla entstammen (Martin J et al., 2012). Das Durchschnittsalter der Patienten bei Diagnosestellung liegt zwischen dem 60. und 70.Lebensjahr. Die Inzidenz an einem Karzinom der Papilla vateri zu erkranken ist in den letzten 30 Jahren stark gestiegen.

Die 5-Jahresüberlebensrate liegt zwischen 34 und 66% (Kohler et al., 2011).

Zu den Symptomen zählen ein Ikterus, Diarrhoe, Steatorrhoe, Gewichtsverlust und Müdigkeit. Als nicht spezifische Symptome können Bauchschmerzen, Übelkeit und Fieber auftreten (Martin et al., 2012).

# Methoden

# Ermittlung des Ernährungszustandes, Anthropometrie

Der Ernährungszustand wird unter anderem mit anthropometrischen Messungen erhoben. Dafür sollen die Körpergröße [m], das Körpergewicht [kg] und der BMI (Body mass index) [kg/m²] genutzt werden.

### BMI (Body mass index)

Der BMI wird aus dem Quotienten von Körpergewicht [kg] und der Körpergröße zum Quadrat [m²] ermittelt.

Die Formel lautet:

BMI [kg/m²]= Körpergewicht [kg]/ Körpergröße [m²]

Nach Ermittlung des BMIs erfolgt die Einteilung der Patienten in eine untergewichtige (BMI<18,5kg/m²), eine normalgewichtige (BMI 18,5 - 24,9kg/m²) und in eine übergewichtige bzw. adipöse Patientengruppe (übergewichtig: BMI >25kg/m² und <30kg/m², adipös: BMI> 30kg/m²).

### Bioelektrische Impedanzanalyse (B.I.A.)

Zur Bestimmung der Körperzusammensetzung soll die Bioelektrische Impedanzanalyse durchgeführt werden, diese ist eine verbreitete, nicht invasive Methode zur Analyse der Körperzusammensetzung. Dem Patienten werden jeweils zwei Elektroden an Hand und Fuß, nach Reinigung der Haut, befestigt und mittels eines Impedanzanalysegeräts (Data Input-MBM 2000) ein schwaches, homogenes, nicht spürbares Stromfeld im Körper erzeugt. Gemessen wird bei einem Wechselstrom von 800mA und 50kHz sowie bei 5kHz.

Während der Messung werden zwei unterschiedliche elektrische Widerstände bestimmt. Zum einen der Wasserwiderstand (R), aus dem das Körperwasser, die Magermasse und das Körperfett bestimmt werden und zum anderen der Zellwiderstand (Xc), welcher die Organ- und Muskelmasse des Körpers anzeigt. Aus diesen Messwerten kann die dazugehörige *Software Nutri Plus* die komplette Körperzusammensetzungsanalyse vornehmen.

### L3-Computertomographie

Für diese Studie wird keine extra CT veranlasst. Die Quantifizierung der Körperzellmasse mit Hilfe von L3 erfolgt nur dann, wenn beim Patienten im Verlauf der routinemäßigen Diagnostik eine CT veranlasst wurde oder der Patient in PANRAD eingeschlossen wurde und somit eine CT vorliegt. Die Auswertung der Bilder erfolgt mittels der Software Osirix.

Der Patient muss zur biphasischen Mehrzeilen CT nüchtern erscheinen. Dem Patienten werden 500ml Wasser oral verabreicht. Anschließend muss der Patient 5 Minuten auf der rechten Seite liegen, damit das Wasser ins Duodenum gelangen kann. Zwei Ampullen Buscopan werden 1:10 verdünnt und intravenös appliziert. Daraufhin wird eine bisphasische Pankreasdoppelspirale mit 100ml Accupaque oder Visipaque iv. mit einer Flussrate von 3ml/sek. appliziert.

### Magnetresonanztomographie

Hat der Patient routinemäßig oder im Verlauf von PANRAD eine MRT erhalten, wird dieses ebenfalls zur Quantifizierung der Körperzellmasse verwendet. Auch externe MRT Untersuchungen können in die Befunderhebung eingeschlossen werden.

Verwendet wird am Klinikum Greifswald folgendes MRT: 1.5 Tesla Magnetom Avanto (Siemens Health Care, Erlangen). Die Untersuchung besteht aus einem standardisierten Sequenzprotokoll (siehe PANRAD). Einschließlich Lagerung, Einstellung und SHIM-Vorgänge werden großzügig zehn Minuten veranschlagt. Eine Kontrastmittelgabe ist nicht erforderlich.

### Optidiet

OptiDiet ist ein Programm mit Fachinformationen und Empfehlungen zu einer Vielzahl von Diäten und Allergien. Die Datenbasis bildet der komplette aktuelle Bundeslebensmittelschlüssel, der durch eine Fülle von diätetischen Lebensmitteln ergänzt ist. Die Nährwertangaben spielen insbesondere bei der Berechnung von Ernährungsprotokollen, Plänen und Rezepten eine große Rolle und ermöglichen eine präzise Nährwertberechnung.

Die Nährstoffempfehlungen für Gesunde richten sich im Programm nach den offiziellen DGE-Empfehlungen und sind als Bewertungskurven abgebildet. Bei den einzelnen Diäten werden diese Bewertungskurven entsprechend den diätetischen Zielen und Vorgaben modifiziert. Datengrundlage für die Diäten bilden die verschiedenen Versionen des Rationalisierungsschemas der Deutschen Gesellschaft für Ernährungsmedizin und zahlreiche Schriften und Bücher über Ernährungsmedizin und Diätetik.

### Messung der klinisch relevanten Parameter

Die Bestimmung des großen Blutbildes, Glucose, C-reaktiven Proteins, der Triglyceride, HDL-Cholesterin, LDL-Cholesterin, Albumin, ALAT, ASAT, GGT, Bilirubin, Kreatinin, Harnstoff, TSH/T_3_/T_4_, Elektrolyte gehört zu den Standard-Laboruntersuchungen und erfolgt durch das Institut für klinische Chemie und Laboratoriumsmedizin der Universitätsmedizin Greifswald.

#### Erfassung der Lebensqualität

*EORTC QLQ-C30*

Der EORTC QLQ-C30 ist ein multidimensionaler Fragebogen, der die Lebensqualität von Krebspatienten erfasst. Dieser setzt sich aus 6 Teilskalen zusammen, die die Leistungsfähigkeit abfragen, sowie 3 Teilskalen und 6 Items, die einzelnen Symptome abfragen. Als Subskalen lassen sich physische Beeinträchtigungen, Rollenverhalten, kognitive Auswirkungen, emotionale Auswirkungen, soziale Beeinträchtigungen, globale Lebensqualität, Erschöpfung, Erbrechen und Übelkeit, Schmerzen sowie weitere symptombezogene Items verwenden.

## Ernährungstherapie

Nach der Ermittlung des Energieumsatzes wird eine supportive Substratzufuhr ausgewählt, um einen Gewichtsverlust einzuschränken bzw. zu verhindern.

Bei der bekanntesten Formel zur Schätzung des Ruheenergieverbrauchs handelt es sich um die Formel von Harris und Benedict, welche zur Berechnung das Körpergewicht, die Körpergröße, das Geschlecht und das Alter berücksichtigt.

*Harris Benedict, 1919:*Für Männer:

REE = 66,473 + (13,752 * Körpergewicht (in kg)) + (5,003 * (Körpergröße in cm)) - (6,755 * Alter in Jahren).

Für Frauen:

REE = 655,096 + (9,5634 * Körpergewicht (in kg)) + (1,850 * (Körpergröße in cm)) - (4,676 * Alter in Jahren). (Harris Benedict, 1919)

*Nutritional Risk Screening (NRS)*

Das Nutritional Risk Screening nach Kondrup ist ein validierter Score zur Erfassung des Risikos für eine Mangelernährung. Dabei wird die Schwere der Erkrankung (metabolische Stresssituation) dem Ernährungszustand gegenübergestellt und eine Punktezahl errechnet, gemäß welcher die Indikation zur Ernährungstherapie gestellt werden kann.

*Subject Global Assessment (SGA)*

Der SGA ist eine einfache durchzuführende und reproduzierbare bedside Methode zur Einschätzung des Ernährungszustandes bei stationären und ambulanten Patienten.

Erfasst werden der Gewichtsverlust, verminderte Nahrungszufuhr, Verlust von subkutanem Fettgewebe, gastrointestinale Symptome und die Beeinträchtigung der Leistungsfähigkeit.

# Studienbeginn

Die Patientenrekrutierung beginnt, sobald das Vorhaben von der zuständigen Ethikkommission positiv begutachtet wurde. Die Studie wird bei clinicaltrials.gov (NCT) angemeldet.

# Studienablauf

| **Studienablauf** | **Screening-Visite**  **Zeitpunkt 0** | **Visite bei Entlassung** | **Follow up**  **Woche 12** |
| --- | --- | --- | --- |
| Klinische Untersuchung | **•** | **•** | **•** |
| Patientenaufklärung | **•** |  |  |
| Ernährungsrelevante Parameter | **•** | **•** | **•** |
| Festlegen der Ernährungstherapie | **•** | **•** | **•** |
| BIA-Messung | **•** | **•** | **•** |
| QLQ | **•** | **•** |  |
| Ermittlung des Energieumsatzes | **•** | **•** | **•** |
| Blutabnahme | **•** | **•** | **•** |
| Ernährungsprotokoll | **•** |  | **•** |
| Fakultativ (wenn vorhanden):  L3-CT quantitativ  MRT | **•**  **•** |  | **•**  **•** |

# Abbruch der Behandlung

Die Studie kann unter folgenden Umständen abgebrochen werden:

1. Auf Wunsch des Patienten – der Patient kann zu jedem Zeitpunkt frei entscheiden aufzuhören.
2. Wenn ein Studienleiter der Meinung ist es wäre nachteilig für den Patienten die Behandlung fortzuführen.

Wenn es bei einem Patienten zum Abbruch kommen sollte, muss der Studienleiter die genauen Gründe dafür dokumentieren. Wenn möglich sollte die Schlussbeurteilung immer komplettiert werden. Allerdings hat der Patient das Recht, sowohl von der gesamten Studie, als auch von bestimmten Teilen zurückzutreten. Wenn der Abbruch aufgrund eines bestimmten Ereignisses stattfindet, sollte dieses sorgfältig dokumentiert und der Patient überwacht werden, bis sich die Situation geklärt hat. Im Falle eines Abbruchs wäre es dennoch gut, wenn die Patienten an der Nachuntersuchung teilnehmen, natürlich nur mit deren Einverständnis.

# Statistische Überlegungen

Studien, die den Einfluss von parenteraler Ernährung auf das Gewicht in Nüchternphasen während des stationären Aufenthaltes untersuchen sind rar.

Den positiven Effekt einer zusätzlichen parenteralen Ernährung auf das Gewicht (bei Patienten mit kolorektalem Karzinom), belegt eine Studie von Hasenberg et al., welche im Jahr 2010 durchgeführt wurde. Der Gewichtsverlust lag bei den Patienten im Durchschnitt bei 7kg in 3 Monaten (±2kg). Bei Patienten mit parenteraler Ernährung blieb der BMI (Body mass index) und die BCM (Body cell mass) konstanter als bei den Patienten, die nur orale Zusatzernährung bekamen. Während der Körperfettanteil der Kontrollgruppe während des Zeitraumes kontinuierlich sank, blieb dieser in der Interventionsgruppe konstant. Bei Patienten in der Kontrollgruppe stieg der Körperwasseranteil.

Eine Poweranalyse unter Verwendung der Standardabweichung aus Hasenberg et al. und einer angenommen Gewichtsdifferenz zwischen Interventions- und Kontrollgruppe von 2 kg ergibt eine nötige Gesamtpatientenzahl von n = 28 bei einer Teststärke von 0,8 bzw. n = 48 bei einer Teststärke von 0,95 (Abbildung 1). Wird von nur 1 kg Gewichtsdifferenz ausgegangen, ergeben sich entsprechend Stichproben von n = 106 bzw. n = 184.

Abbildung 1: Abhängigkeit der benötigten Stichprobengröße von der Teststärke

Die statistische Auswertung der Daten erfolgt mit STATA 11. Für alle untersuchten Parameter wird eine beschreibende Statistik erstellt. Der Vergleich der Patienten, die eine parenterale Supplementierung in Nüchternphasen bekommen, mit denen, die Standard care erhalten, erfolgt mit Hilfe des nichtparametrischen Mann-Whitney-U-Tests für zwei unabhängige Stichproben.

Zum Vergleich von zwei Methoden (BIA mit L3-CT oder MRT) wird ein Bland-Altman-Diagramm verwendet.

# Ethik und Datenschutz

Die Patientendaten werden in anonymisierten Erhebungsbögen erfasst und digital in Computern der Ernährungsmedizin gespeichert.

Die Bilddatensätze werden für die Analyse anonymisiert, verbleiben aber im PASC-System der Radiologie.

Die elektronische Speicherung der Daten erfolgt pseudonymisiert unter einem Identifikationscode. Alle Daten der Materialsammlung werden kennwortgeschützt auf einem Computer der Inneren Medizin A der Uniklinik Greifswald zentral gespeichert. Zugriff auf nicht anonymisierte Daten haben nur die an der Studie beteiligten Mitarbeiter.

# Ergebnisse

Patienten, die zur Abklärung einer fokalen Läsion des Pankreas oder der Gallenwege in die Klinik kommen sind häufig Nüchternphasen ausgesetzt. Während dieser Zeit kann es zu einem Gewichtsverlust kommen. Mithilfe der parenteralen Supplementierung soll der Gewichtsverlust verhindert/verringert werden und bei bereits eingetretener Mangelernährung eine Verbesserung des Ernährungszustandes angestrebt werden.

Aufgrund der Ergebnisse der Studie von Hasenberg et al. 2010 gehen wir davon aus, dass eine parenterale Supplementierung während des stationären Aufenthaltes einen positiven Effekt für die Patienten hat. Wir vermuten, dass die Patienten keinen/einen geringeren Gewichtsverlust erleiden, dass die Körperzusammensetzung konstant bleibt (Körperfettanteil, BCM, Gesamtkörperwasser, Zellanteil und Phasenwinkel) und sich das Outcome der Patienten verbessert. Hasenberg et al. (2010) konnten in ihrer Studie zeigen, dass Patienten mit einem kolorektalem Karzinom von einer parenteralen Ernährung profitieren. Die Patienten, welche eine parenterale Zusatzernährung bekamen, konnten ihr Gewicht/BMI länger konstant halten. Der Gewichtsverlust lag bei ca.7kg (±2kg) in drei Monaten. Da die Patienten im Durchschnitt eine Woche zur Diagnosestellung in die Klinik kommen, gehen wir davon aus, dass Patienten ohne eine parenterale Zusatzernährung im Durchschnitt 1-2kg Gewicht verlieren.

Die vorliegende Studie soll überprüfen, ob eine parenterale Supplementierung während des stationären Aufenthaltes zu einer besseren Lebensqualität führt als bei Pateinten mit Flüssigkeitssubstitution.

Ebenfalls sollen die Ergebnisse der Bioelektrischen Impedanzanalyse mit den Ergebnissen der L3-CT zur Quantifizierung der Körperzellmasse miteinander verglichen werden. Da die CT im Moment als gute Methode zur Darstellung der Körperzellmasse gilt, gehen wir davon aus, dass die Bioelektrische Impedanzanalyse ähnliche oder schlechtere Ergebnisse erzielt.

# Literaturliste

Correia M.I.; Waitzberg D.L.: *The impact of malnutrition on morbidity, mortality, length of hospital stay and costs evaluated through a multivariate model analysis*. Clinical Nutrition, **2003**. 22(3): 235-239

Fearon K.C.H., Baracos V.E.: Cachexia in pancreatic cancer: new treatment options and measures of success. International Hepato-Pancreato-Biliary Association, **2010**.12, 323-324

Fearon K.C.H.: Cancer cachexia and Fat-Muscle Physiology. The New England Journal of Medicine **2011**. 365; 6;565-567

Gaßler N, Knüchel R.**:**Springer-Verlag (2012) **DOI:** 10.1007/s00292-011-1546-8

Grant J.P: *Nutritional Support in Acute and Chronic Pancreatitis*. Surgical Clinical North America 91 **2011** 805-820

Gray C., MacGillivray T.J., Eeley C., Stephens N.A., Beggs I., Fearon K.C.and Greig C.A.: *Magnetic resonance imaging with k-means clustering objectively measures whole muscle volume compartments in sarcopenia/cancer cachexia*. Clinical Nutrition **2011**. 30:106-111

Harris JA, Benedict FG. *A Biometric Study of Basal Metabolism in Man*.

Washington, DC: Carnegie Institute; **1919**. Publication No. 279.

Hasenberg T.; Essenbreis M.; Herold A.; Post S. and Shang E.: *Early supplementation of parenteral nutrition is capable of improving quality of life, chemotherapy-related toxicity and body composition in patients with advanced colorectal carcinoma undergoing palliative treatment: results from a prospective, randomized clinical trial.* Colorectal disease, **2010**. 12, e190-e199

Khan S.A., Toledano M.B. and Taylor-Robinson D.: *Epidemiology, risk factors, and pathogenesis of cholangiocarcinoma***. 2008**. HPB, 10:77-82

Kohler I., Jacob D., Budzies J., Lehmann A., Weichert W., Schulz S., Neuhaus P.and Röcken C.: *Phenotypic and Genotypic Characterization of Carcinomas of the Papilla of Vater has Prognostic and Putative Therapeutic Implications***.2011** American Journal of Clinical Patholoy.135:202-211

Lim J.H.: Cholangiocarcinoma: *Morphologic Classification According to Growth Pattern and Imaging Findings*. AJR.2003.181:819-827

Martin J.A, Moser A.J, Howell D., Travis A.C. and Savarese: *Ampullary carcinoma: Epidemiology, clinical manifestations, diagnosis and staging.* **2012**

McWhirter J.P. and Pennington C.R.: *Incidence and recognition of malnutrition in hospital*. BMJ **1994**; 308:945-8

Meier R.F.: *Nutrition in pancreatic diseases*. **2006** Best Practice & Research Clinical Gastroenterology. Vol.20, No.3, pp 507-529

Mitsiopoulos N., Baumgartner R.N., Heymsfield S.B., Lyons W., Gallagher D. and Ross R.: Cadaver validation of skeletal muscle measurement by magnetic resonance imaging and computerized tomography. The American Physiological Society, **1998.** 115-122

Pirlich M; Schütz T; Norman K; Gastell S; Lübke H J; Bischoff S C; Bolder U; Frieling T; Güldenzoph H; Hahn K; Jauch K-W; Schindler K; Stein J; Volkert D; Weimann A; Werner H; Wolf C; Zürcher G; Bauer P; Lochs H: *The German hospital malnutrition study*. Clinical nutrition, **2006**.25:563-572

Reissfelder, C.; Koch M., Büchler M.W.; Weitz J.: *Pankreaskarzinom*. Chirurg **2007**. 78:1059-1072

Rosen C., Nagorney D., Wiesner R., Coffey R., La Russo N.: *Cholangiocarcinoma complicating primary sclerosing cholangitis* 1991.Ann Surg 213,1 p. 21-5

Sharma, C.; Eltawil, K.M.; Renfrew Paul D.; Walsh, M.J.; Molinari M.: *Advances in diagnosis, treatment and palliation of pancreatic carcinom*a: *1990-2010.* World Journal of Gastroenterology, **2011** February 21; 21 17(7):867-897

Vincent A; Herman J.; Schulick R.; Hruban R.H.; Goggins M.: *Pancreatic Cancer*. Published online May 26, **2011** www.thelancet.com
