# Supplementary material for: Early Parenteral Nutrition in Patients with Biliopancreatic Mass Lesions, a Prospective, Randomized Intervention Trial
Source: PLoS One. 2016 Nov 18;11(11):e0166513. doi: 10.1371/journal.pone.0166513 (PMC5115751; doi:10.1371/journal.pone.0166513)
Supplement: S1 Table — (DOCX) [file pone.0166513.s005.docx]

**S1 Table:** **Reported complications in the intervention and control group during hospital stay.**

| Type of complication | Control (n=49) | Intervention (n=51) |
| --- | --- | --- |
| Infection (thrombophlebitis) | 0 | 1 (1.96%) |
| Hyperglycemia | 3 (6.12%) | 4 (7.84%) |
| Hypokalemia | 1 (2.04%) | 0 (0%) |
| Hypertension | 3 (6.12%) | 2 (3.92%) |
